# Effect of Dry and Water-Slurry Dosing of Different Silica Fumes on the Mechanical Properties of Standard Mortar

**DOI:** 10.3390/ma19183987

**Published:** 2026-09-19

**Authors:** Grzegorz Rogojsz, Tomasz Rudnicki

**Affiliations:** Faculty of Civil Engineering and Geodesy, Military University of Technology, 2 Gen. Sylwestra Kaliskiego Str., 00-908 Warsaw, Poland; tomasz.rudnicki@wat.edu.pl

**Keywords:** silica fume, slurry dosing, dry dosing, standard mortar, compressive strength, bending strength, particle size, agglomeration

## Abstract

Microsilica is a commonly used additive in cementitious materials due to its high amorphous SiO_2_ content, very small particle size, and high pozzolanic activity. However, its effectiveness may depend not only on the chemical composition and physical properties of the material but also on the method of its incorporation into the cement mixture. The aim of the study was to evaluate the effect of dry and slurry dosing of three microsilicas with different chemical compositions and grain size characteristics on the consistency and mechanical properties of standard mortar. White microsilica (MKb), compacted microsilica (MZ), and Mikrosill+ microsilica (MK+) were used in the study at concentrations of 1%, 3%, 5%, and 7% by weight of cement. In the slurry dosing method, the microsilica was added to part of the mixing water, and the prepared slurries were conditioned for three days before sample preparation. Material properties were assessed based on particle size distribution analysis and SEM microscopic observations, while mortar properties were assessed based on consistency and bending and compressive strength after 7, 28, and 56 days of curing. The obtained results showed that the effect of dosing method was not clear and depended on the type of microsilica, its content, sample age, and the type of strength tested. In the case of compressive strength, the dosing in the form of an aqueous suspension was more favorable in many systems after 7 days, while after 56 days, in most cases, higher values were obtained for dry-dosed microsilicas. In the case of bending strength, more favorable results were generally obtained at a younger age for dry-dosed microsilicas, while after 56 days, this relationship was less clear. Granulometric analysis revealed differences in the formation and disintegration of agglomerates of the tested microsilicas upon contact with water. The results indicate that the effectiveness of the microsilica dosing method cannot be assessed independently of the properties of the specific material and the mortar maturation age.

## 1. Introduction

The cement industry plays a fundamental role in the development of modern infrastructure, but it is also a major source of anthropogenic CO_2_ emissions. Portland cement production involves energy-intensive clinker production and calcination processes, which significantly contribute to the carbon footprint of cement-based materials. Therefore, reducing clinker consumption while maintaining the required mechanical properties and durability remains an important challenge for the construction sector [1,2].

One way to reduce the environmental impact of cementitious materials is to partially replace Portland cement with supplementary cementitious materials (SCMs). Properly selected mineral additives reduce clinker content and can also positively impact the properties of fresh and hardened cement mixtures. Depending on their chemical composition and physical properties, these additives can exhibit hydraulic or pozzolanic activity, or act primarily through physical filling. Their use can reduce porosity, modify the microstructure, and improve the mechanical and durability properties of cementitious materials [3,4].

A wide range of mineral additives have been analyzed in recent decades, including fly ash, ground granulated blast furnace slag, limestone powder, meta-kaolin, natural pozzolans, glass powder, basalt dust, and microsilica [5,6]. The use of the above mineral additives has a positive effect on strength parameters, increasing compressive strength from 5% to 15% and at the same time allowing for the cement content to be reduced by up to 20% [7].

Among the various mineral additives, microsilica has received particular attention due to its unique physical and chemical properties. Microsilica is an ultrafine-grained material produced as a byproduct during the production of silicon metal and ferrosilicon alloys in electric arc furnaces. It is characterized by a high content of amorphous silicon dioxide and a very small particle size, which translates into a large specific surface area and high potential pozzolanic activity [8,9]. The introduction of microsilica into cementitious materials affects both the hydration process and the development of microstructure. Amorphous silica can react with calcium hydroxide, leading to the formation of additional C–S–H products [10]. At the same time, very fine microsilica particles can act as microaggregate, improving the tightness of the aggregate stack of the mineral mixture and limiting the size of capillary spaces. The combination of both effects is considered one of the primary factors responsible for the beneficial properties of cement concretes containing microsilica [11]. Numerous studies have shown that the use of microsilica can lead to increased compressive and bending strength and to reduced water permeability and absorption [7,12,13,14]. Microsilica studies often analyze the additive content at a few percent by weight of cement. Increasing the microsilica content leads to improvement in the properties of cement concrete [15]; however, it can also increase water demand and affect the consistency of the mix. Therefore, not only is the amount of additive used important, but also its distribution within the cement matrix [16]. The effectiveness of microsilica depends not only on its chemical composition and pozzolanic activity, but also on particle size, specific surface area, tendency to agglomerate, and the ability to achieve appropriate dispersion during mixing. These parameters determine the actual accessibility of the particle surface for reactions occurring in the cement paste [17,18]. Due to its very small particle size and high specific surface area, microsilica has a strong tendency to form agglomerates. These secondary particle clusters can behave like larger structures during mixing, limiting the material surface area directly accessible to the cementitious environment. Consequently, the actual effectiveness of microsilica may depend on the degree of fragmentation and the distribution of agglomerates in the mix. This phenomenon was investigated by the authors of [19,20]. Microsilica is available in various forms, including bulk material, compacted material and in the form of an aqueous suspension [21]. The individual forms differ in their transport properties and behavior during mix preparation. Of particular importance in this case is the way in which microsilica agglomerates are dispersed during mixing. Introducing the material as an aqueous suspension can alter the state of aggregation and the distribution of the particles before they make contact with the cement. The authors of [22] demonstrated that microsilica dosed in dry form exhibits better dispersion in mortar. Previous studies indicate that the degree of microsilica dispersion can influence the development of the microstructure, mechanical properties, and transport properties of concrete mixtures. However, the use of an aqueous suspension cannot be unequivocally equated with achieving better dispersion, as the behavior of microsilica in water depends on its physical and chemical properties and degree of compaction [23,24]. Only a limited number of studies directly compare different methods of introducing microsilica into the cement system. The authors of [25,26,27,28] compared the use of microsilica in the form of an aqueous and dry suspension and demonstrated that both modification methods can positively influence the properties of cement concretes, although the differences between the analyzed methods were not clear. It should also be noted that the above studies analyzed compacted microsilica dosed at 8% to 10% by weight of the binder. The authors focused primarily on studies of cement concretes also containing coarse aggregate with a grain size of up to 20 mm. These results indicate that the form of microsilica delivery alone does not necessarily have to be a factor that clearly determines the final properties of the material.

Despite there being extensive research on microsilica, relatively little attention has been paid to directly comparing dry and slurry dosing methods while maintaining the most comparable material preparation conditions. The number of studies conducted on standard mortars, which help reduce the impact of variations in aggregate composition and properties and enable direct comparison of the material’s mechanical properties, is particularly limited. Therefore, it remains unclear whether the differences between dry and slurry dosing methods result primarily from the physical form of the additive or from changes in the state of particle agglomeration and dispersion.

Taking into account our own experience and the fact that adding microsilica in amounts of 10% and 20% to cement mortars improved their strength properties, the authors decided to test whether a lower microsilica content in cement mortar would also positively impact strength parameters. Additionally, they decided to determine whether the method of dosing microsilica in the form of a suspension in cement mortar would have a similar effect to using this type of dosing in cement concrete. Therefore, the aim of this study was to evaluate the effect of dry and slurry dosing of three microsilicas—white microsilica (MKb), compacted microsilica (MZ), and Mikrosill+ microsilica (MK+)—on the consistency and mechanical properties of a standard mortar. The analysis was conducted for four levels of cement substitution with microsilica, 1%, 3%, 5%, and 7%, and three maturation periods: 7, 28, and 56 days. Additionally, the particle size distribution and morphology of the tested materials were assessed, with particular emphasis on changes occurring upon contact with water.

## 2. Materials and Methods

### 2.1. Materials

In order to assess the influence of microsilicas and their application method on the strength properties of the standard mortar, standard sand from KWARCMIX, Tomaszów Mazowiecki, Poland, which meets the requirements specified in the standard, was used in laboratory tests [29]; additionally, cement class CEM I 42.5 R from CEMEX, Warszawa, Poland was used, hereinafter referred to as CEM I, and the parameters are presented in Table 1.

**Figure 1:**
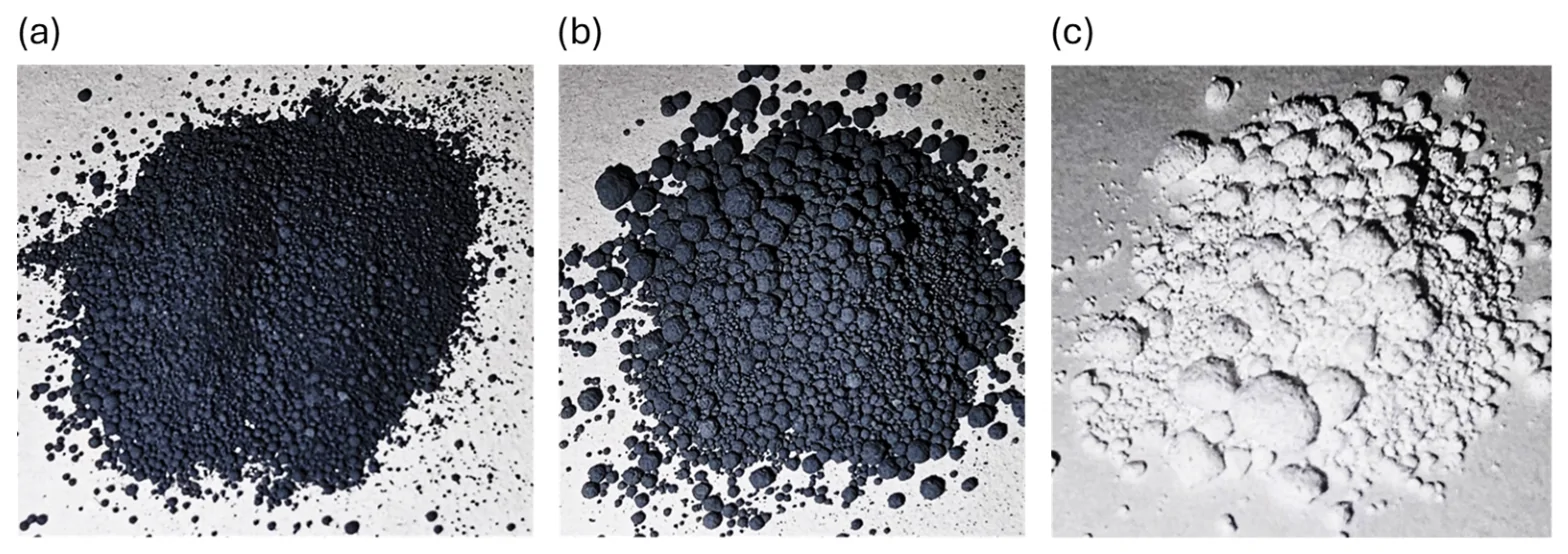
(**a**) Compacted microsilica, (**b**) Mikrosill+ microsilica, and (**c**) white microsilica.

**Figure 2:**
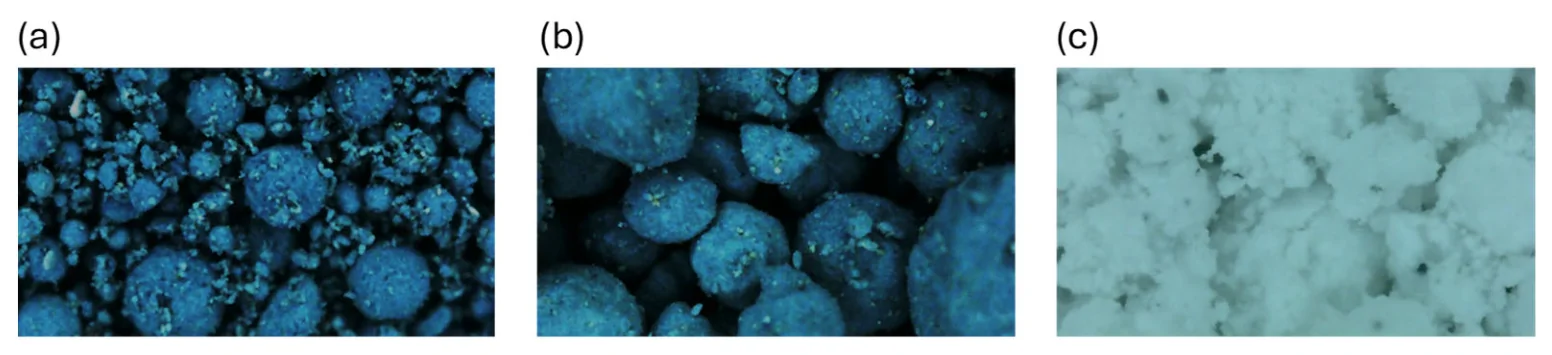
Photos magnified at 50×: (**a**) compacted microsilica, (**b**) Mikrosill+ microsilica, and (**c**) white microsilica.

**Figure 3:**
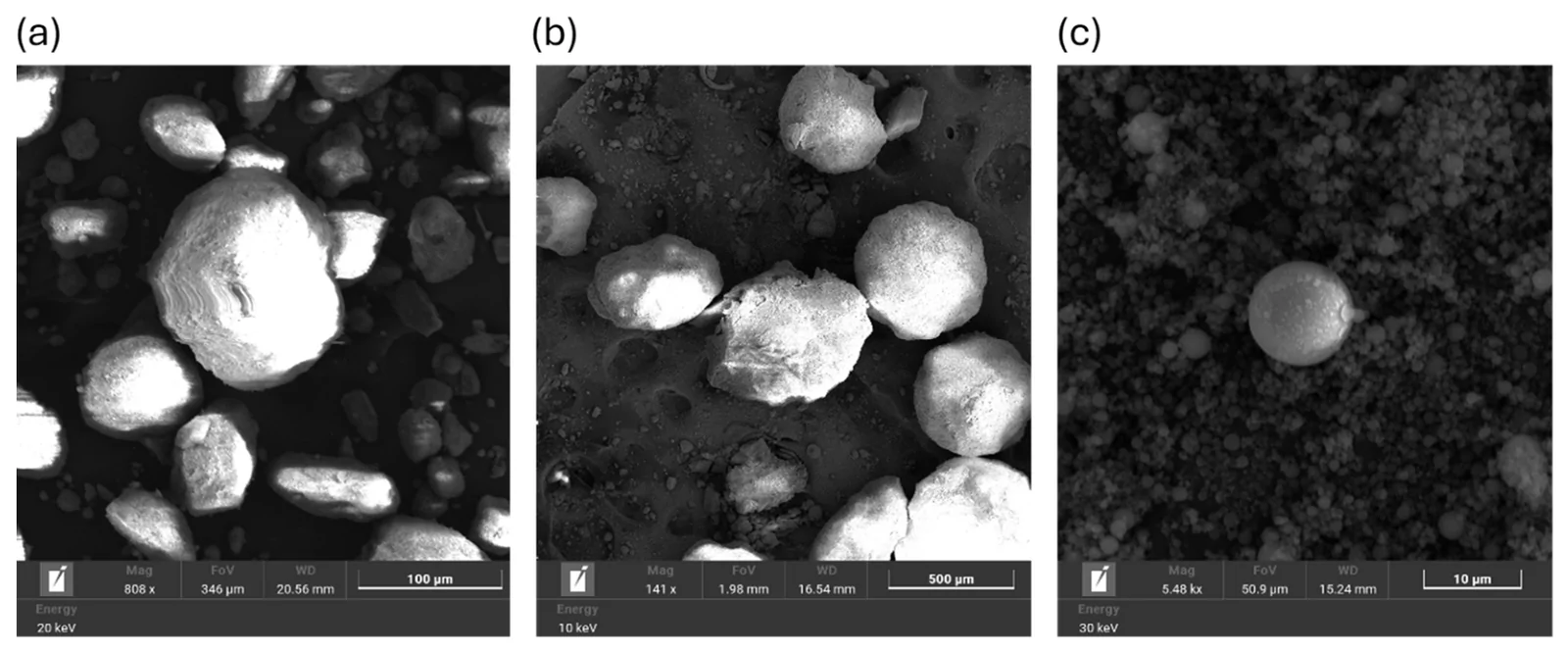
SEM images: (**a**) compacted microsilica, (**b**) Mikrosill+ microsilica, and (**c**) white microsilica.

Microsilicas from Mikrosilika Trade, Stalowa Wola, Poland, were used in laboratory tests: white microsilica (MKb), compacted microsilica (MZ), and Mikrosill+ microsilica (MK+). White microsilica is a byproduct of zirconium silicate production, while compacted microsilica and Mikrosill+ microsilica are produced in electric arc furnaces during the production of metallic silicon and ferrosilicon alloys. The materials used differed in chemical composition, which may affect their behavior when in contact with water and in cementitious systems. The chemical composition of the microsilicas used, as declared by the manufacturer, is presented in Table 2, and illustrative photos are provided in Figure 1, Figure 2 and Figure 3.

**Figure 4:**
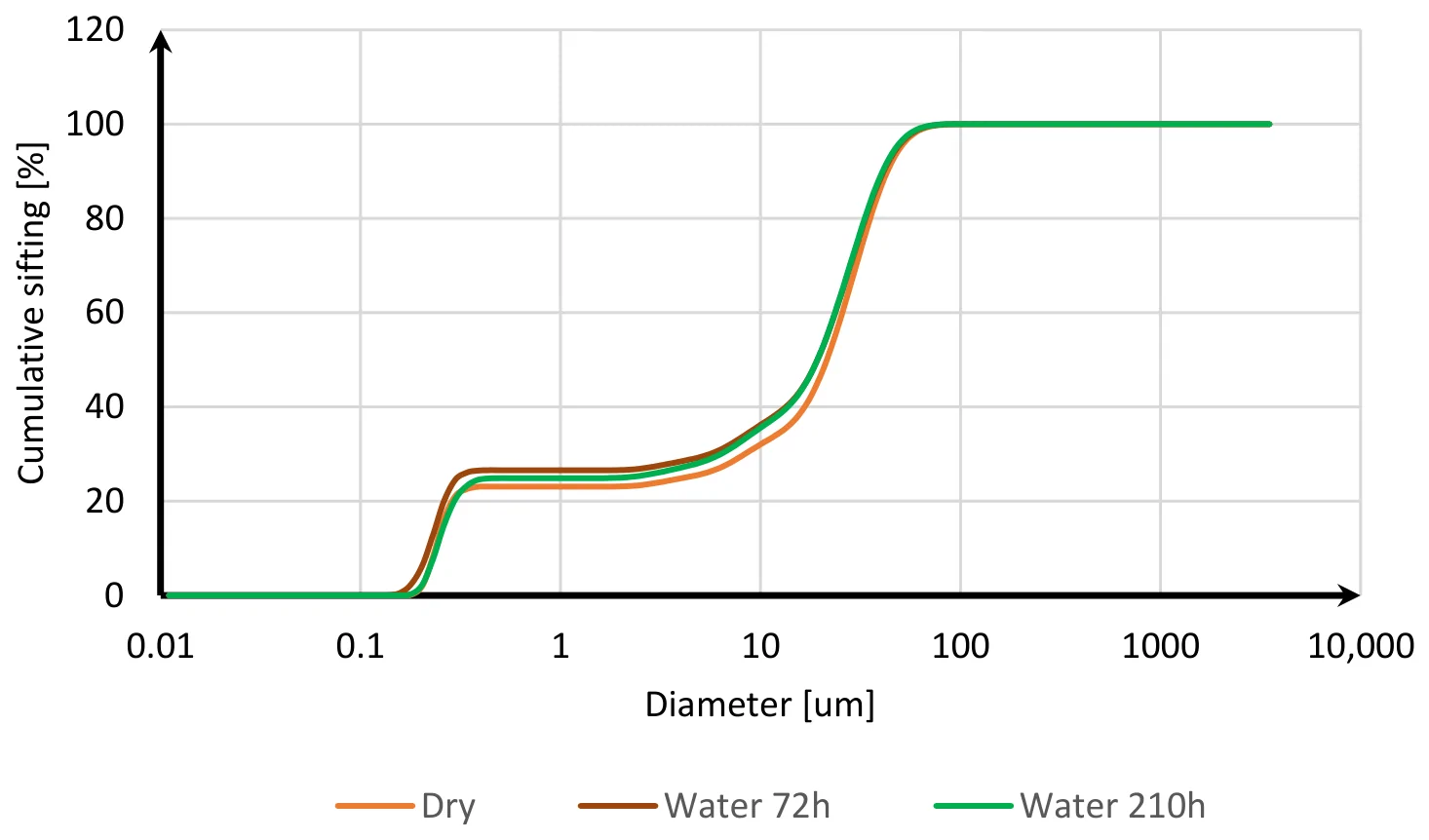
Cumulative grain size curve of compacted microsilica.

**Figure 5:**
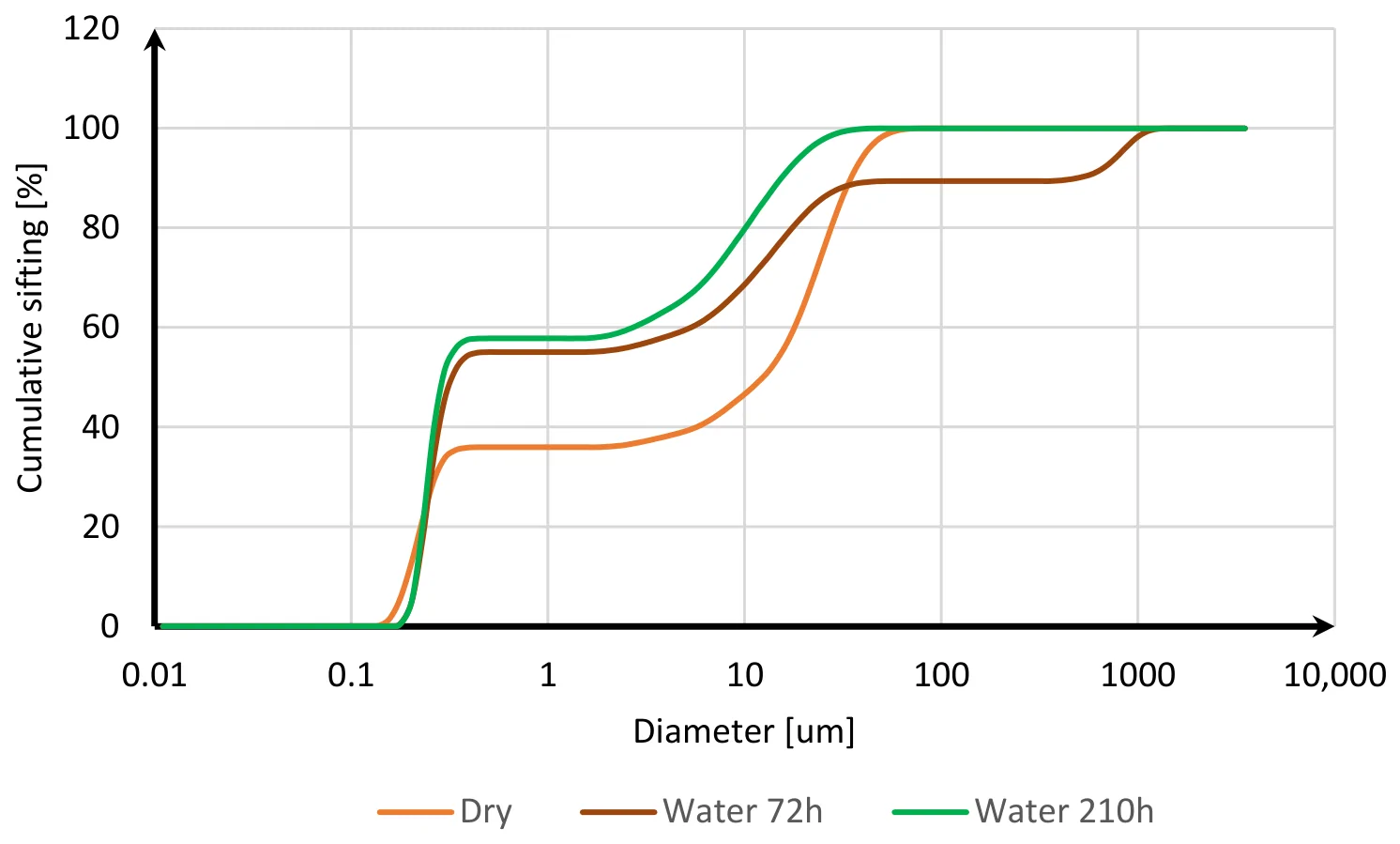
Cumulative grain size curve of Mikrosill+ microsilica.

**Figure 6:**
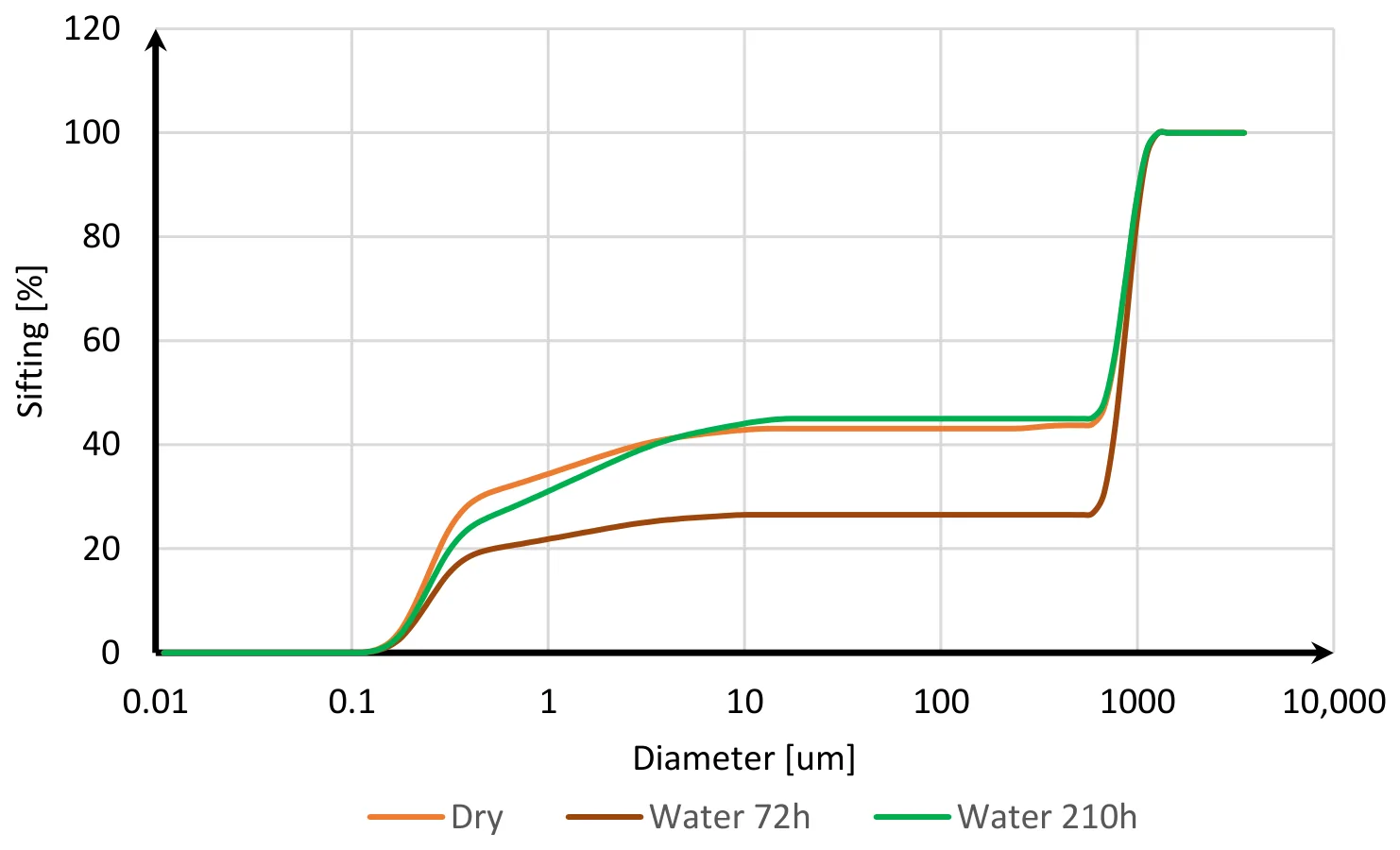
Cumulative grain size distribution curve of white microsilica.

Since microsilicas can be incorporated into mortars both dry and as an aqueous suspension, their behavior when in contact with water was analyzed. Particle size distribution and specific surface area were determined using a Bettersizer S3 plus Laser Particle Size Analyzer, Bettersize Instruments Ltd. from Dandong, China. Distilled water was used as the dispersion medium. Due to the small sample mass of 10 mg used in a single measurement, three measurements were performed for each microsilica and each water contact time. The material was analyzed in its dry state and after 72 and 210 hours of conditioning the microsilicas in water. To measure the particle size of the water-conditioned samples, 5 g of each microsilica was placed in a 20 ml container and covered with distilled water. The samples were conditioned at room temperature for 72 and 210 hours before testing. The results are presented in Figure 4, Figure 5 and Figure 6, while the characteristic parameters of particle size distribution and specific surface area are summarized in Table 3.

For compacted microsilica (MZ), the particle size distribution curves for the dry material and samples after 72 and 210 hours of contact with water remained practically unchanged. The D50 value decreased from approximately 21.4 µm to 19.2 µm, while the D90 value decreased from 42.84 µm to approximately 41 µm. Such small changes indicate that under the tested conditions, contact with water did not alter the observed particle size distribution. SEM images obtained using a VEGA Compact scanning electron microscope (Tescan, Brno, Czech Republic) show grains with a relatively uniform morphology, a similar degree of sphericity, and a limited number of secondary connections between particles.

In the case of Mikrosill+ microsilica (MK+), the material in both the dry and wet states was characterized by a finer particle distribution than MZ. In the case of Mikrosill+ microsilica, it is clearly visible that with prolonged contact with water, the particle size decreases. The D50 value decreased from 12.4 μm to 0.3 μm, while the D90 value decreased from 34.7 μm to 15.4 μm. SEM images, similar to those for compacted microsilica, show grains with a relatively uniform morphology and a similar degree of sphericity.

In the case of white microsilica (MKb), an unusual situation occurred. Dry samples and those conditioned for 210 hours in water exhibited very similar particle distributions, as confirmed by the distribution shown in Figure 6 and the D50 and D90 values in Table 3. Samples conditioned for 72 hours in water showed larger agglomerates, differing markedly from these results. It should also be noted that the micrographs reveal numerous, nearly spherical primary particles with diameters of around 10 micrometers. During sample preparation, a strong tendency for particles to adhere to each other was observed, hindering their complete separation. This indicates that a significant portion of the material may have existed as secondary agglomerates during the measurement.

This phenomenon is reflected in the results of the granulometric analysis of white microsilica. High D50 values, approximately 718 µm for dry samples and 711 µm after 210 h of contact with water, and D90 values, approximately 1015 µm for dry samples and 1006 µm after 210 h of contact with water, do not necessarily correspond to the sizes of individual particles observed by SEM, but may reflect the size of agglomerates formed as a result of interactions between particles.

The obtained results are consistent with the latest literature reports on the behavior of microsilica in aqueous systems. Mao et al. [30] demonstrated that laser diffraction particle size distribution analysis primarily describes the size of agglomerates present in the suspension, while SEM observations enable the identification of the primary particles that constitute these structures. The authors also demonstrated that the degree of microsilica densification has a decisive influence on the size of the agglomerates, and that sufficiently intense sonication leads to almost complete deagglomeration of the material, resulting in a marked reduction in the D50 value. These results indicate that high values of granulometric parameters do not necessarily indicate the presence of large primary particles but may reflect the stability of secondary agglomerates.

Analysis of the obtained results shows that for compacted and Mikrosill+ microsilica, the granulometric distribution is very similar to that observed after 210 hours of conditioning. The only discrepancy was observed for white microsilica, for which the granulometric distribution after 72 hours of conditioning in water was the least favorable, meaning that microsilica was characterized by the greatest aggregation. Despite this phenomenon, the authors decided to use a three-day conditioning period in further work, which was considered optimal in relation to the granulometric distribution and the test time.

### 2.2. Research Methods

In order to assess the influence of microsilicas and the method of their application on the strength properties of the standard mortar, a set of samples was prepared for laboratory testing in accordance with the procedure described in the standard in [29], in the form of 40 mm × 40 mm × 160 mm bars, with nine used for each measurement test. The required cement content, i.e., 450 g, was replaced with microsilica granules at 1%, 3%, 5%, and 7% by weight of cement. In the first stage of the study, the microsilica granules were added in dry form, and in the second stage of the study, they were added as an aqueous suspension. To dose the suspension, 100 g of the total mixing water was used to prepare the silica fume suspension. The suspension was stored at room temperature for 3 days and homogenized for 15 minutes in a bottle turner immediately before adding it to the mortar. After adding the suspension to the mortar mixer, the container was rinsed with the remaining mixing water to ensure quantitative silica fume transfer. The total water content in each mixture was constant. In order to compare the effect of individual microsilicas, their content, and application method, a reference mortar designated as Z0 was prepared. The composition of individual mixtures for stages 1 and 2, along with the sample symbols used, is presented in Table 4.

Research into the physical properties of mineral mortars containing silica fume—added in either dry form or as an aqueous suspension—began with the determination of their consistency. To this end, a comparative procedure was employed based on the Vicat pin penetration method, which is used to assess the consistency of cement pastes in accordance with EN 196-3 [31]. The tests were conducted using a Vicat apparatus equipped with a measuring pin with a diameter of 10.0 ± 0.05 mm and a measuring container with an internal diameter of 75 ± 10 mm and a height of 40.0 ± 0.2 mm. The procedure employed was not treated as a direct application of the EN 196-3 requirements for mortars, but rather as a method enabling a comparison of the consistencies of the tested mixtures. Bending and compressive strength tests were carried out on previously prepared bars with dimensions of 40 mm × 40 mm × 160 mm in accordance with the procedure described in the standard [29]. Strength tests were carried out after 7, 28 and 56 days of maturing of the mortar samples. The bending strength test was performed on 9 samples for each series, while the compressive strength test was performed on 18 halves remaining after the bending strength test.

## 3. Results

### 3.1. Testing the Consistency of Mixtures

**Figure 7:**
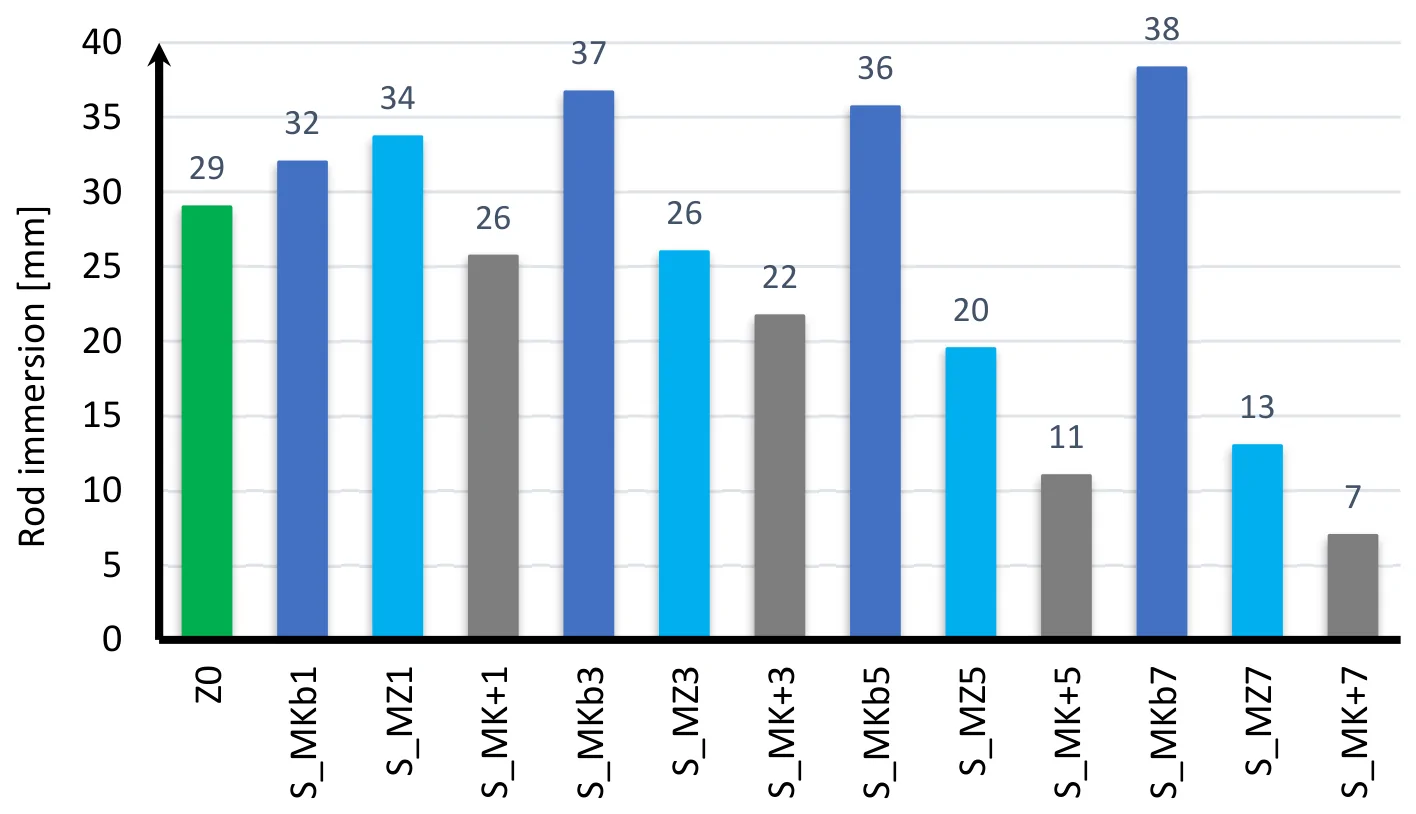
Consistency test results for microsilicas added in dry form.

**Figure 8:**
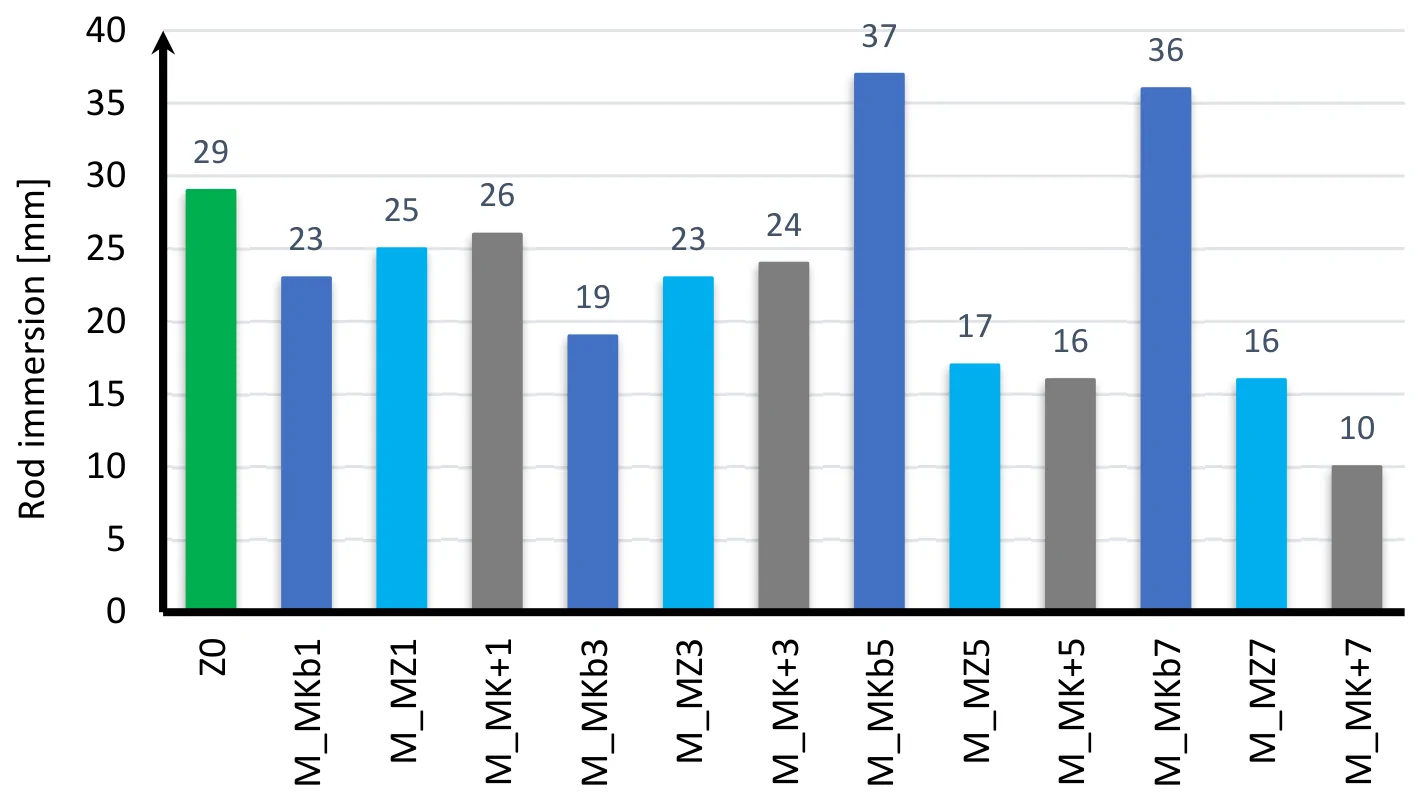
Consistency test results for microsilicas added in suspension form.

The results of the consistency measurement for the mortar with microsilica additives added in dry form are presented in Figure 7, and those for microsilica in wet form are presented in Figure 8.

Based on the presented results, it can be stated that the use of microsilica has a distinct effect on the consistency of standard mortar. In the case of the addition of dry microsilicas, only white microsilica contributed to an increase in mortar consistency. This increase is approximately 27%. The 1% addition of compacted microsilica is also an exception. In the remaining cases, consistency decreases with the microsilica content, to 44% for compacted microsilica and 24% for Mikrosill+ microsilica compared to the baseline samples. This relationship appears to be correct due to the fact that compacted microsilica and Mikrosill+ microsilica are finer than cement and therefore have a higher water demand, so as their content increases, the mixture becomes drier. When microsilicas are added as a water suspension, a decrease in mortar consistency can also be observed with an increasing additive content. The consistencies of mixtures with microsilica added in dry form and as a suspension in water are very similar for most microsilicas. The exceptions are 1% and 3% additions of white microsilica which, in the form of an aqueous suspension, contributed to decreases of 20.7% and 34.5% in mortar consistency, respectively. A 1% addition of compacted microsilica in the form of a suspension also contributed to a decrease of 13.8% in mortar consistency.

Analyzing the above results, it can be concluded that the microsilica application method does not clearly affect the consistency of the mixture. Comparing the two dosing methods, similar consistency values were obtained for half of the samples, with the difference in results not exceeding 15%. However, for the other half of the samples, the difference in consistency between the dosing methods ranged from 26% to 49%. The greatest discrepancy in results was observed between the white microsilica dosing methods at 3%, and the smallest difference was observed for the Mikrosill+ microsilica dosing method at 1%.

### 3.2. Bending Strength Test

**Figure 9:**
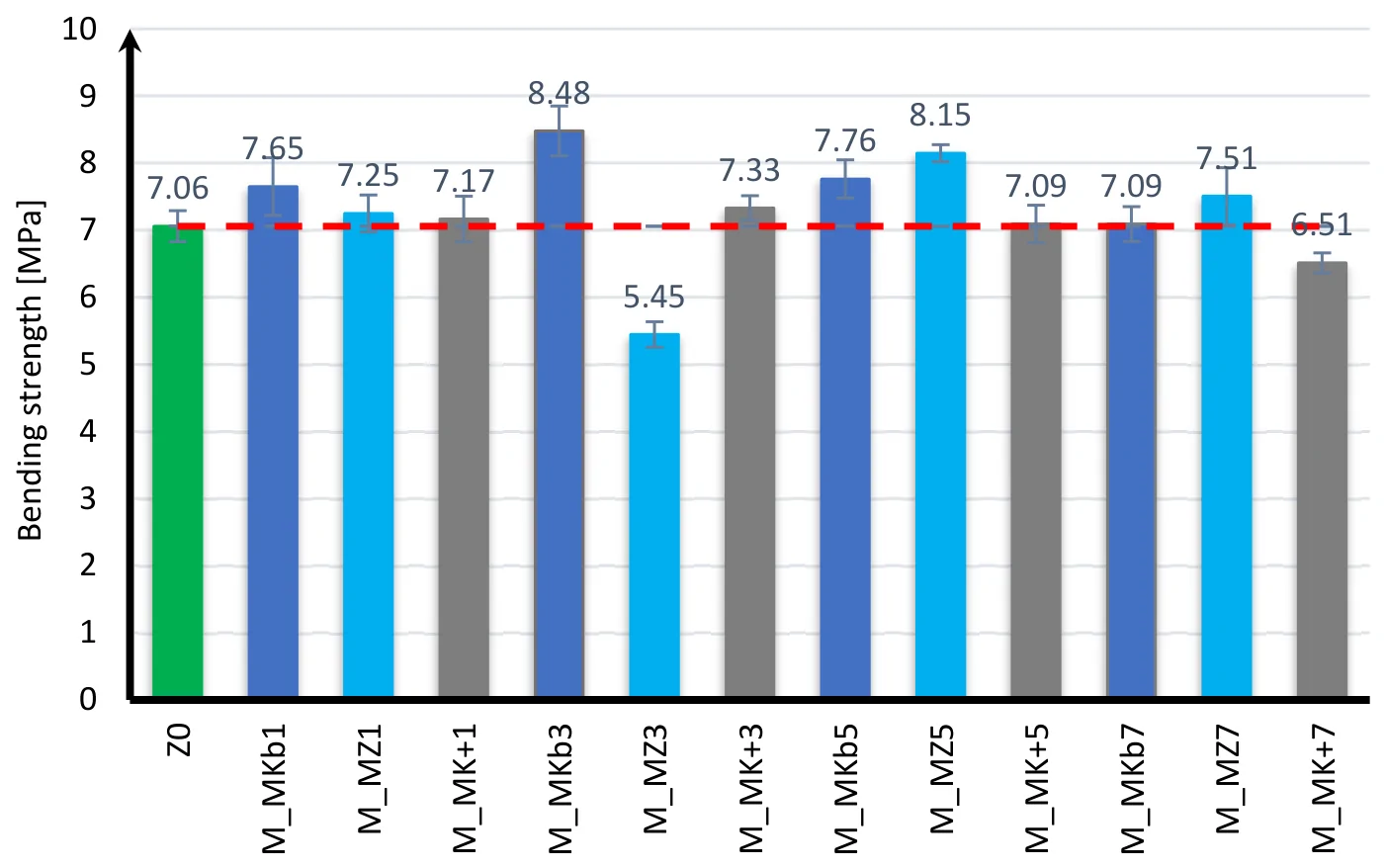
Bending strength after 7 days of conditioning for samples with the addition of dry microsilica.

**Figure 10:**
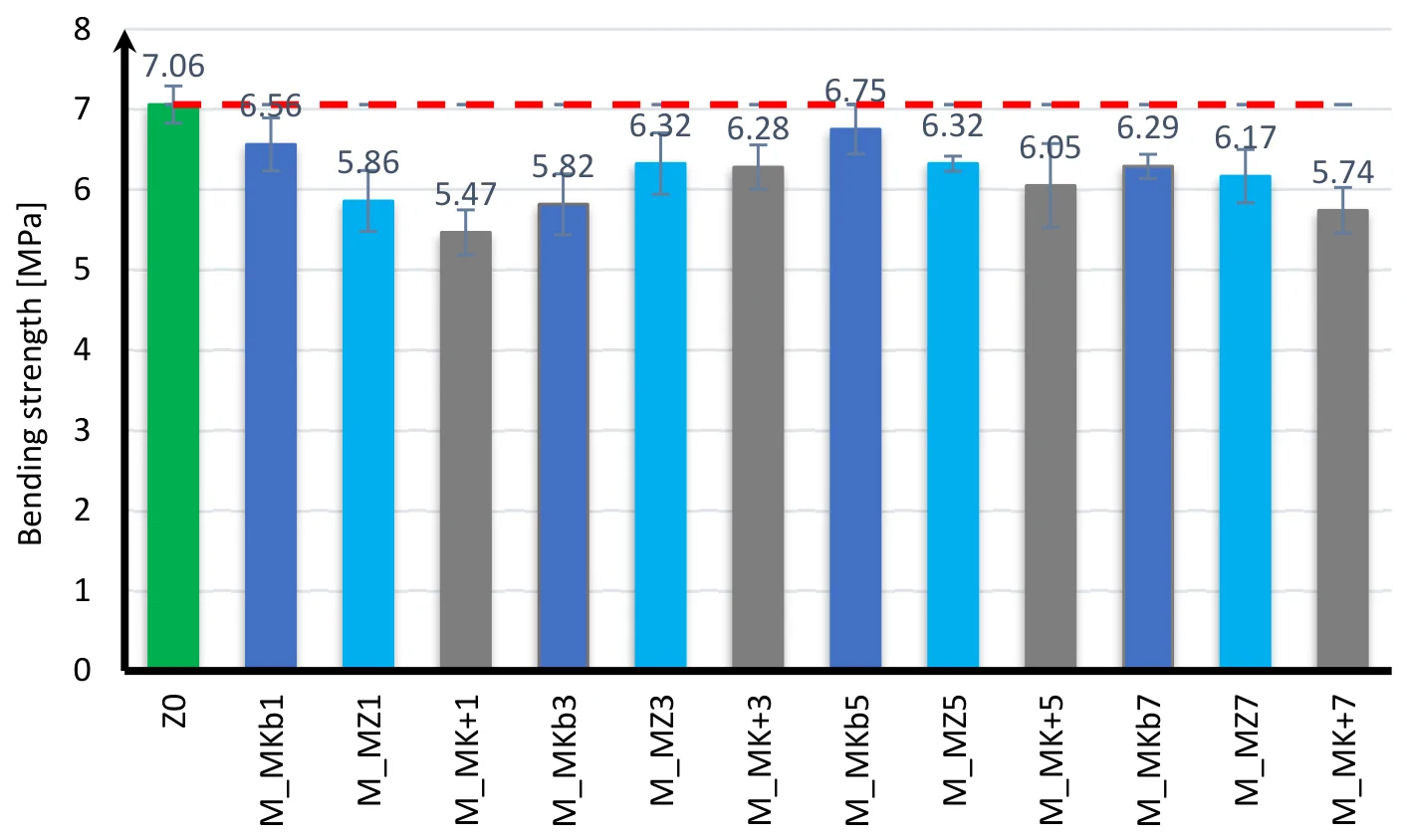
Bending strength after 7 days of conditioning for samples with the addition of microsilica in the form of a suspension.

**Figure 11:**
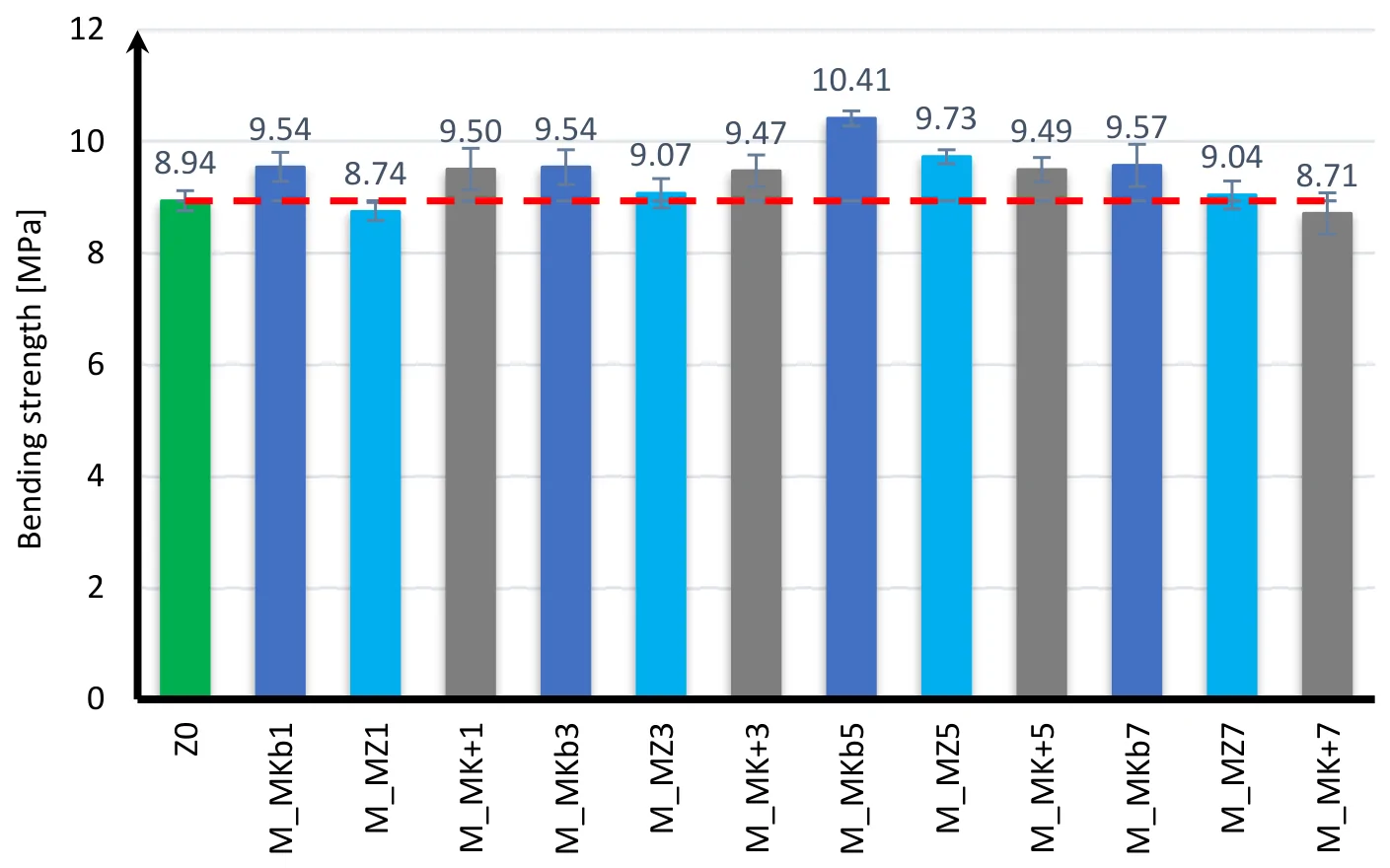
Bending strength after 28 days of conditioning for samples with the addition of dry microsilica.

**Figure 12:**
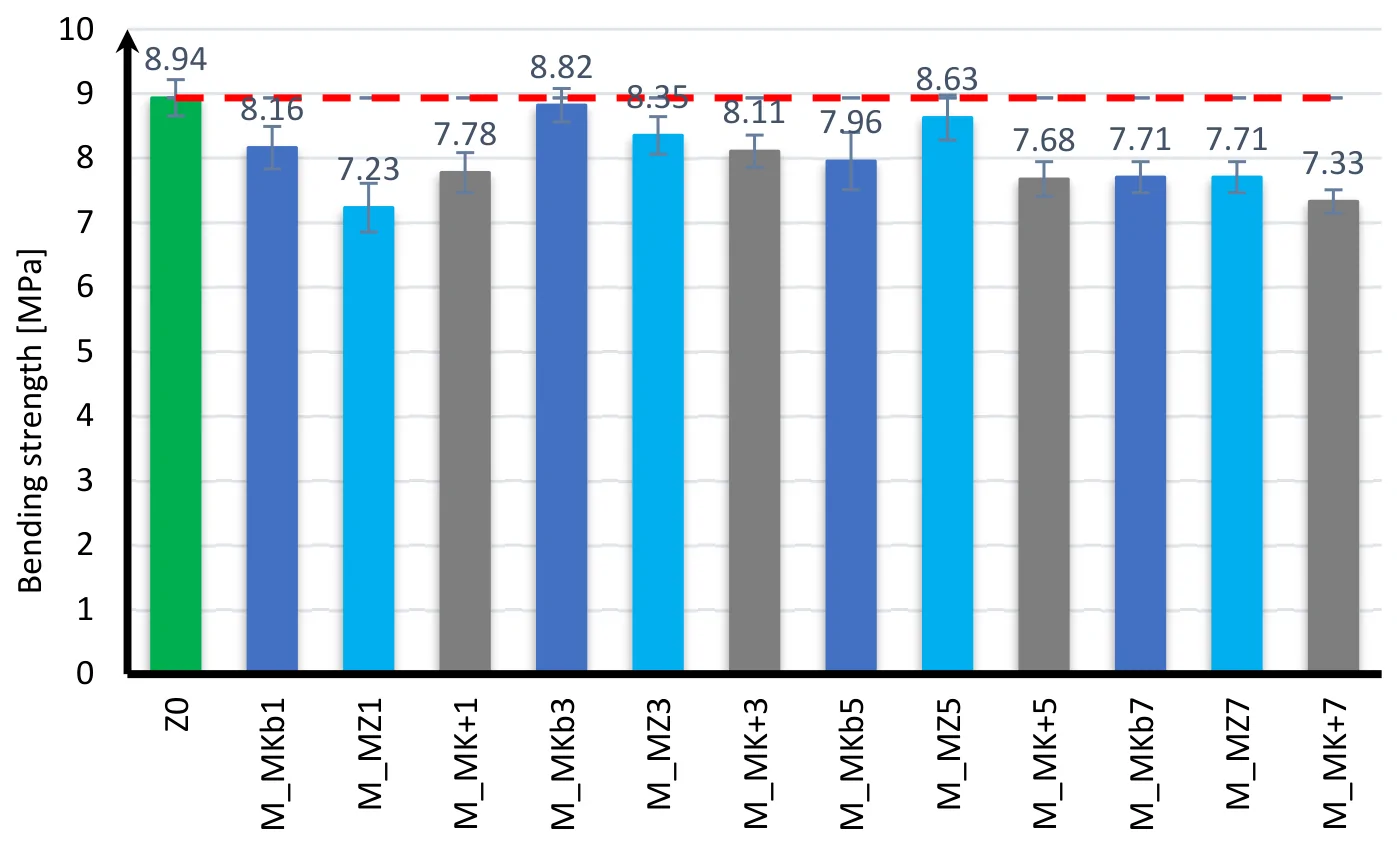
Bending strength after 28 days of conditioning for samples with the addition of microsilica in the form of a suspension.

**Figure 13:**
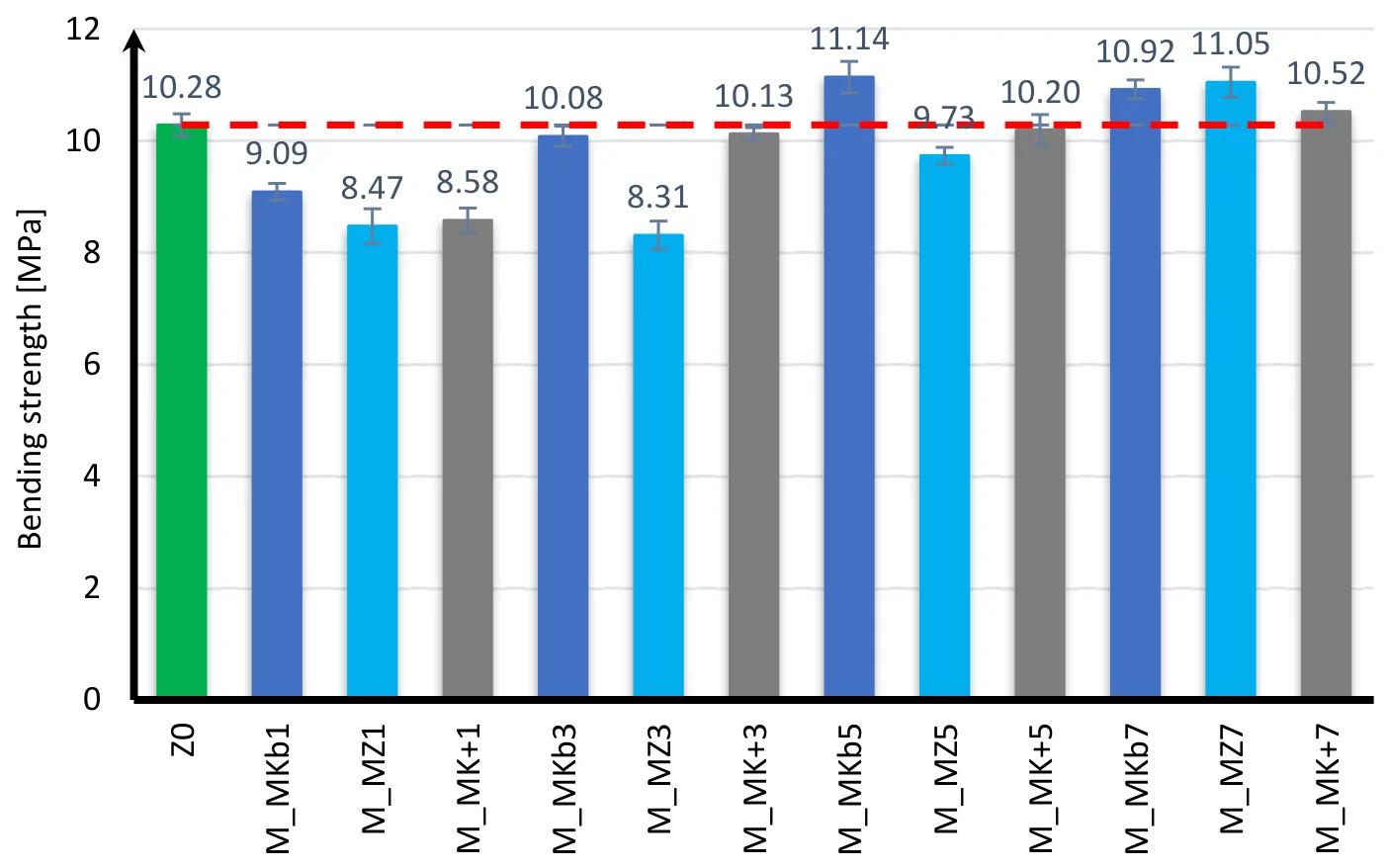
Bending strength after 56 days of conditioning for samples with the addition of dry microsilica.

**Figure 14:**
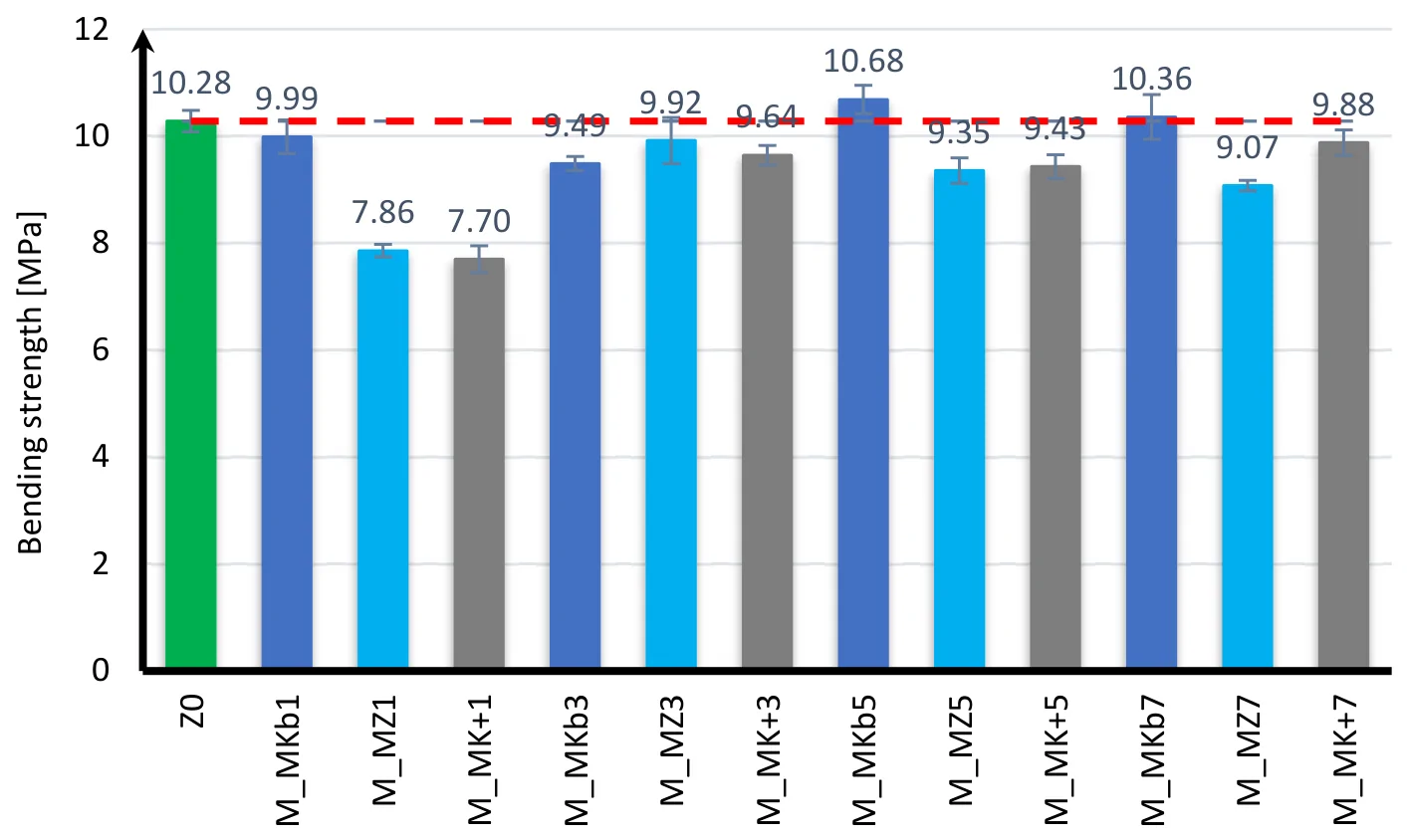
Bending strength after 56 days of conditioning for samples with the addition of micro-silica in the form of a suspension.

Bending strength tests of mortar samples were performed after 7, 28, and 56 days of curing. The bending strength test results after 7 days of conditioning are presented in Figure 9 and Figure 10; those after 28 days of conditioning are presented in Figure 11 and Figure 12; and those after 56 days of conditioning are presented in Figure 13 and Figure 14. The figures show the average bending strength results obtained from measurements of nine samples per series, along with the standard deviation.

Based on the analysis of the obtained results, it can be concluded that the addition of dry microsilica has a positive effect on bending strength results after seven days of conditioning, while the addition of microsilica as an aqueous suspension has a negative effect. In the case of dry microsilica, only a 3% addition of compacted microsilica and a 7% addition of Mikrosill+ microsilica adversely affected the bending strength after seven days of conditioning. The highest strength was achieved by samples with a 3% addition of white microsilica, which reached 120% of the strength of the base samples. White microsilica performed best with 1% and 3% additives, while with 5% and 7% additives, compacted microsilica exhibited greater strength. Based on the bending strength results for microsilica added as a suspension, all samples had lower strengths than those without any additives. The lowest strength, 77.5% of the base sample strength, was achieved by samples with 1% Mikrosill+ microsilica, while the highest strength, 95.6% of the base sample strength, was achieved by samples with 5% white microsilica.

The situation is similar for the bending strength results after 28 days of conditioning. In this case, almost all samples with microsilica added in a dry form exhibited higher strengths than the base samples, while samples with microsilica added in a suspension exhibited lower strengths than the base samples. In the case of the addition of microsilica in a dry form, only the 1% addition of compacted microsilica and the 7% addition of Mikrosill+ microsilica adversely affected the bending strength after 28 days of conditioning. The highest strength was achieved by samples with a 5% addition of white microsilica, which reached 116% of the strength of the base samples. In the bending strength tests after 28 days of conditioning, the samples with white microsilica exhibited the highest strength for each additive content. It can also be concluded that all microsilicas with a 5% addition of white microsilica exhibited the highest strength among those analyzed. Based on the bending strength results for microsilica added as a suspension, all samples were characterized by lower strength than the base samples. The lowest strength, 80.9% of the strength of the base samples, was achieved by samples with 1% compacted microsilica, while the highest strength, 98.6% of the strength of the base samples, was achieved by samples with 3% white microsilica.

When analyzing the bending test results after 56 days of curing, it is impossible to clearly determine whether the microsilica added in dry form or as a suspension has a positive or negative effect on strength. When analyzing the results presented for the dry microsilica addition, it can be seen that all microsilicas with a 7% content have higher strength than the base samples. The highest strength was achieved by samples with a compacted microsilica addition, which was 107.5% of the strength of the base samples. However, the highest strength was achieved by samples with a 5% addition of white microsilica, whose strength was 108.4% of the strength of the base samples. Samples with a 3% addition of white and Microsill+ microsilica, and with a 5% addition of Mikrosill+ microsilica, exhibited strength comparable to the base samples. The remaining samples achieved lower strengths than the base samples; the lowest strengths were found in samples with a 3% addition of compacted microsilica, which achieved 80.8% of the strength of the base samples. When microsilica was added as a suspension, only the samples with a 5% addition of white microsilica achieved strengths 3.9% higher than those of the base samples. Samples with a 7% addition of white microsilica achieved strengths comparable to those without additives. The remaining samples had lower strengths than the base samples. The lowest strengths were found in samples with a 1% addition of compacted and Mikrosill+ microsilica, which achieved strengths of 76.5% and 74.9%, respectively, of the samples without additives.

**Figure 15:**
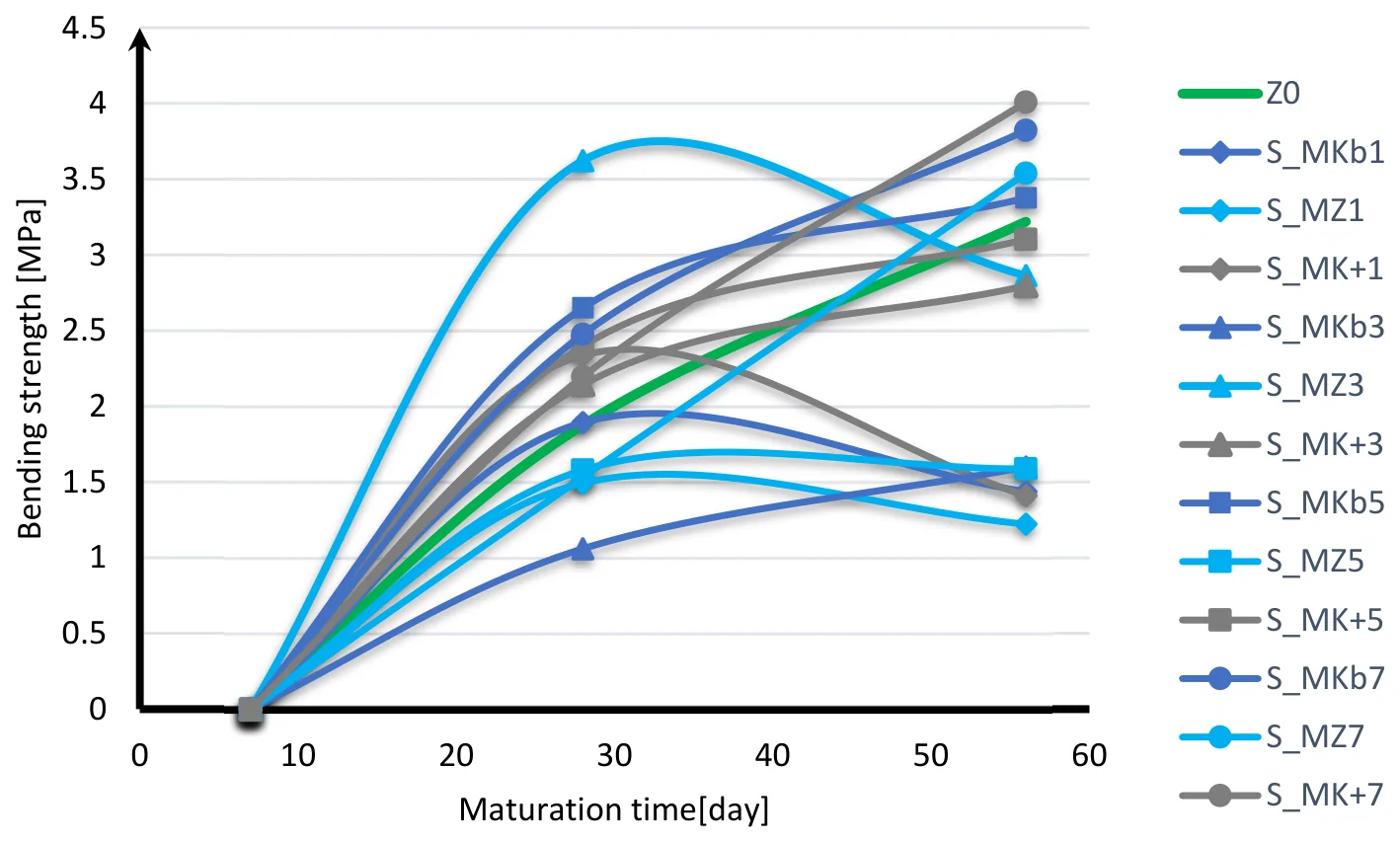
The increase in bending strength over time for samples with the addition of dry microsilica.

**Figure 16:**
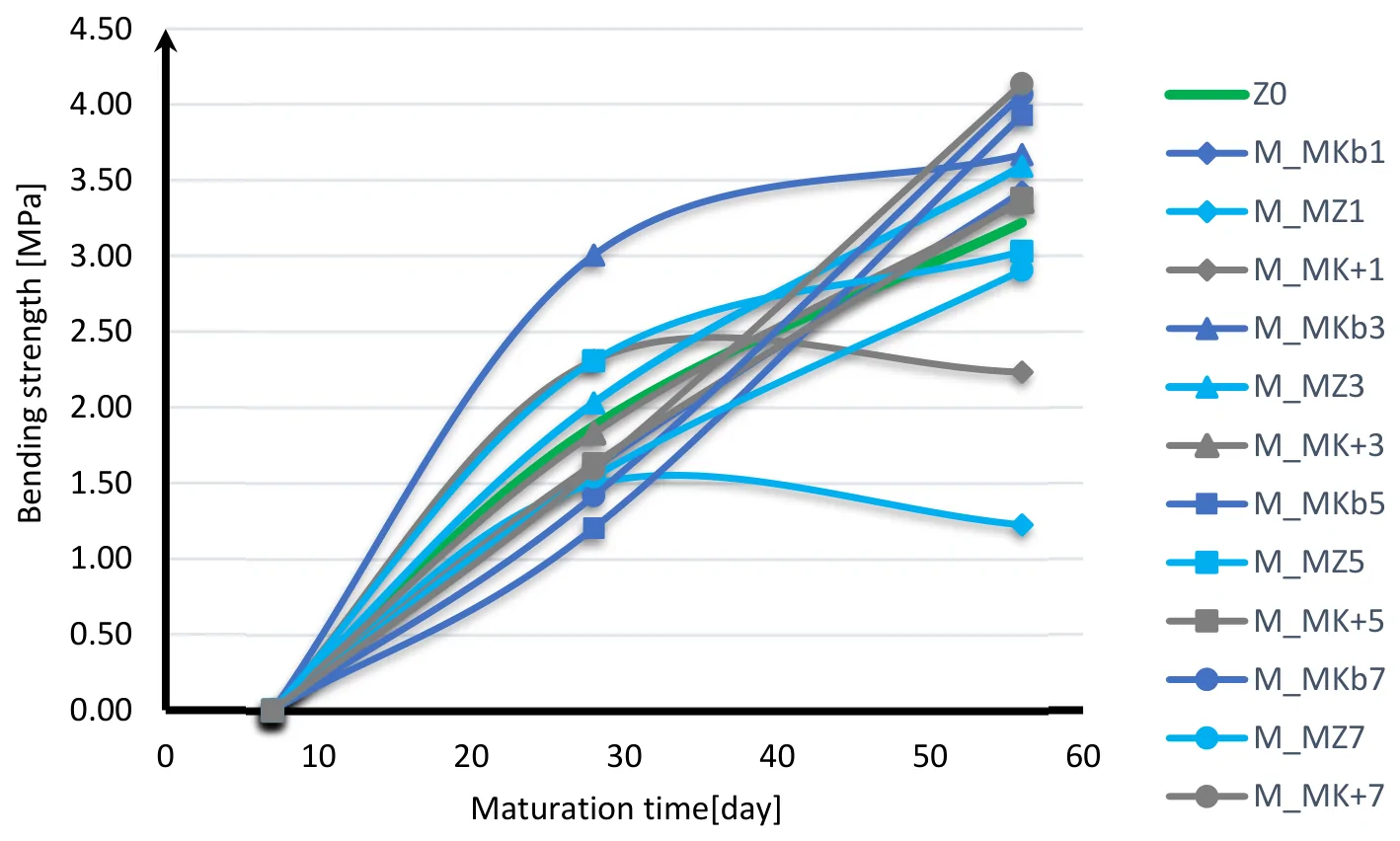
The increase in bending strength over time for samples with the addition of microsilica in the form of a suspension.

When analyzing the results of bending strength increase over time, as presented in Figure 15 and Figure 16, it should be noted that with the addition of dry microsilica, most samples exhibit a faster strength increase between days 7 and 28 of conditioning compared to the baseline samples. The largest initial strength increase was observed in samples with a 3% content of compacted microsilica, which was almost twice as high as in samples without the additive. Samples with the remaining content of compacted microsilica exhibited a weaker strength increase compared to samples without the microsilica additive. It can also be observed that samples with white microsilica at all concentrations had a favorable effect on early strength increase. An unfavorable effect occurred with the 1% content of each microsilica, which achieved lower strength after 56 days of conditioning than after 28 days. This phenomenon was observed for all measured samples. A decrease in strength between 28 and 56 days of conditioning was also observed for the 3% compacted microsilica content, which amounted to 8.4%. When microsilica was added as an aqueous suspension, most samples showed a linear increase in strength similar to that of the base samples. An increase in strength was observed between 28 and 56 days of curing for samples with 5% and 7% white microsilica, and between 7 and 28 days of conditioning for samples with 3% white microsilica. It should also be noted that samples with 1% compacted and Mikrosill+ microsilica achieved lower strengths after 56 days of curing than after 28 days of curing. 

### 3.3. Compressive Strength Test

**Figure 17:**
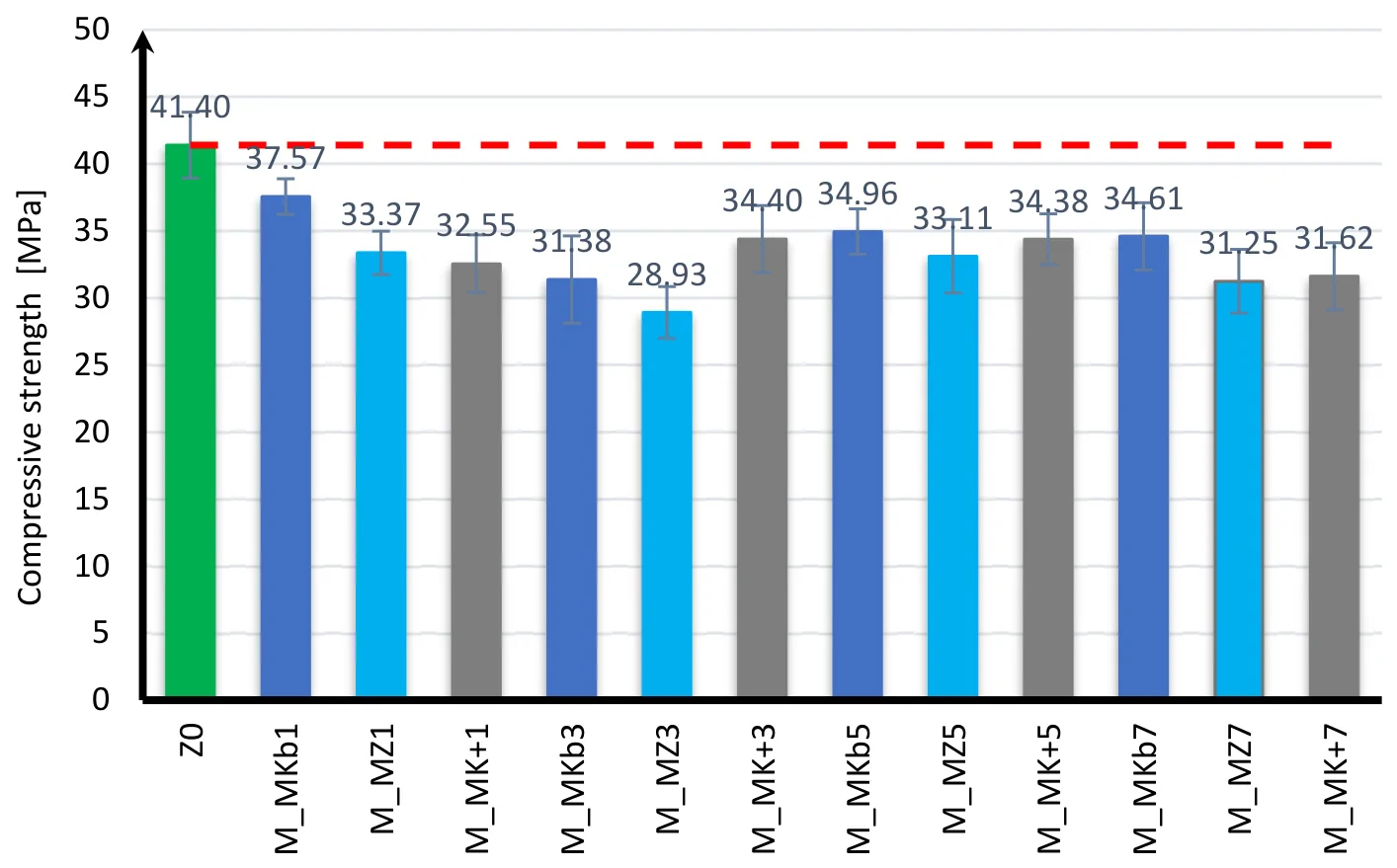
Compressive strength after 7 days of conditioning for samples with the addition of dry microsilicas.

**Figure 18:**
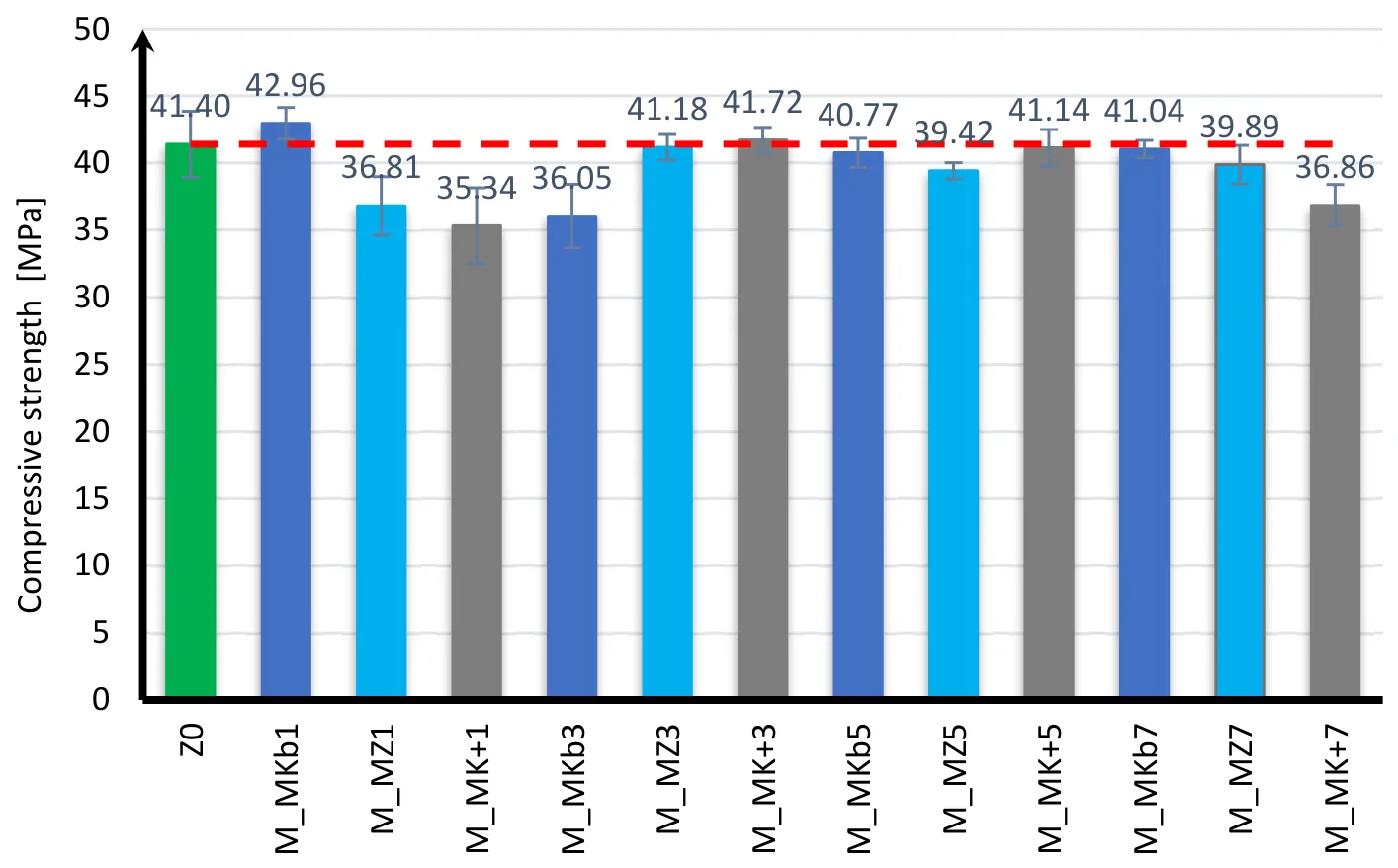
Compressive strength after 7 days of conditioning for samples with the addition of microsilica in the form of a suspension.

**Figure 19:**
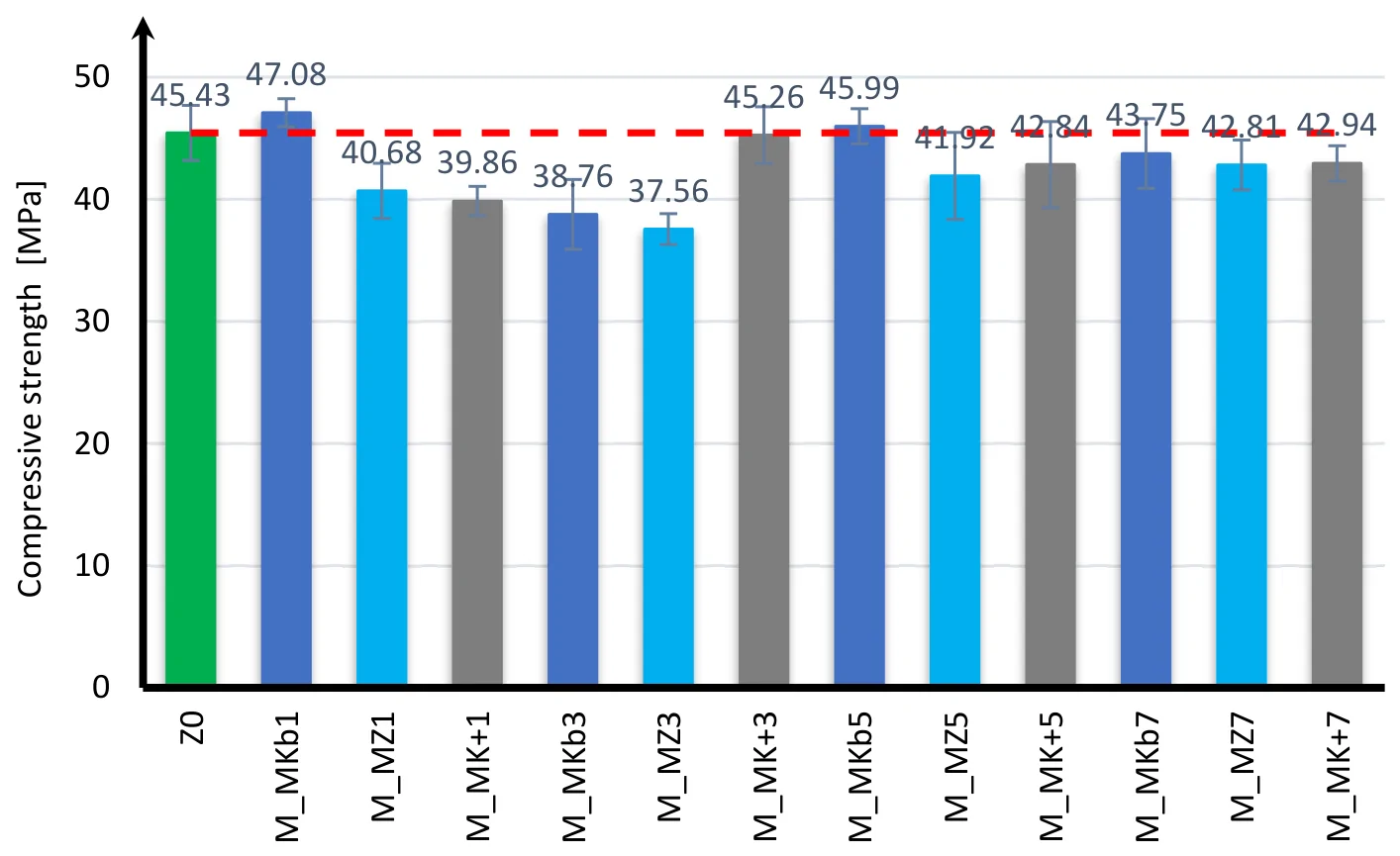
Compressive strength after 28 days of conditioning for samples with the addition of dry microsilica.

**Figure 20:**
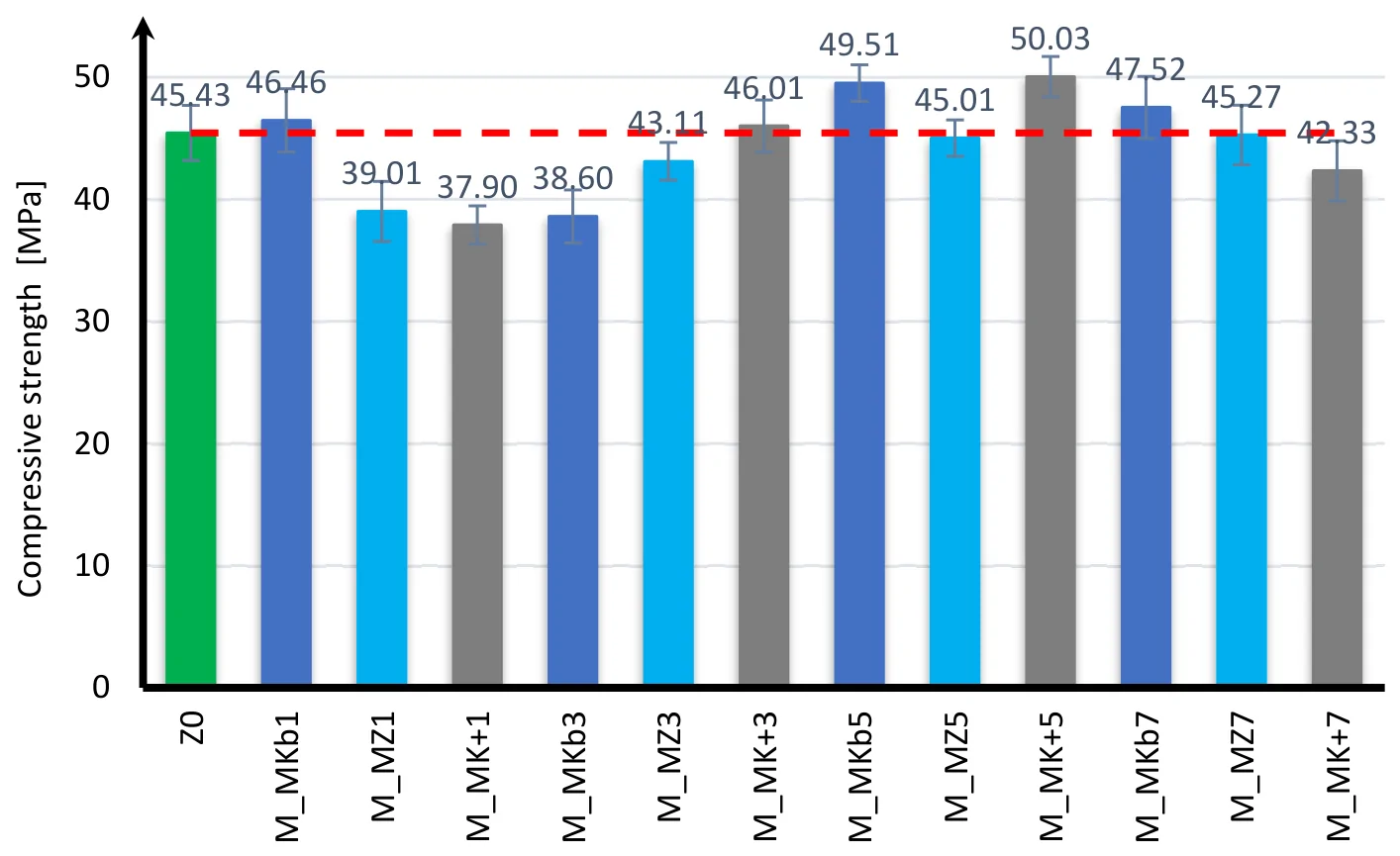
Compressive strength after 28 days of conditioning for samples with the addition of microsilica in the form of a suspension.

**Figure 21:**
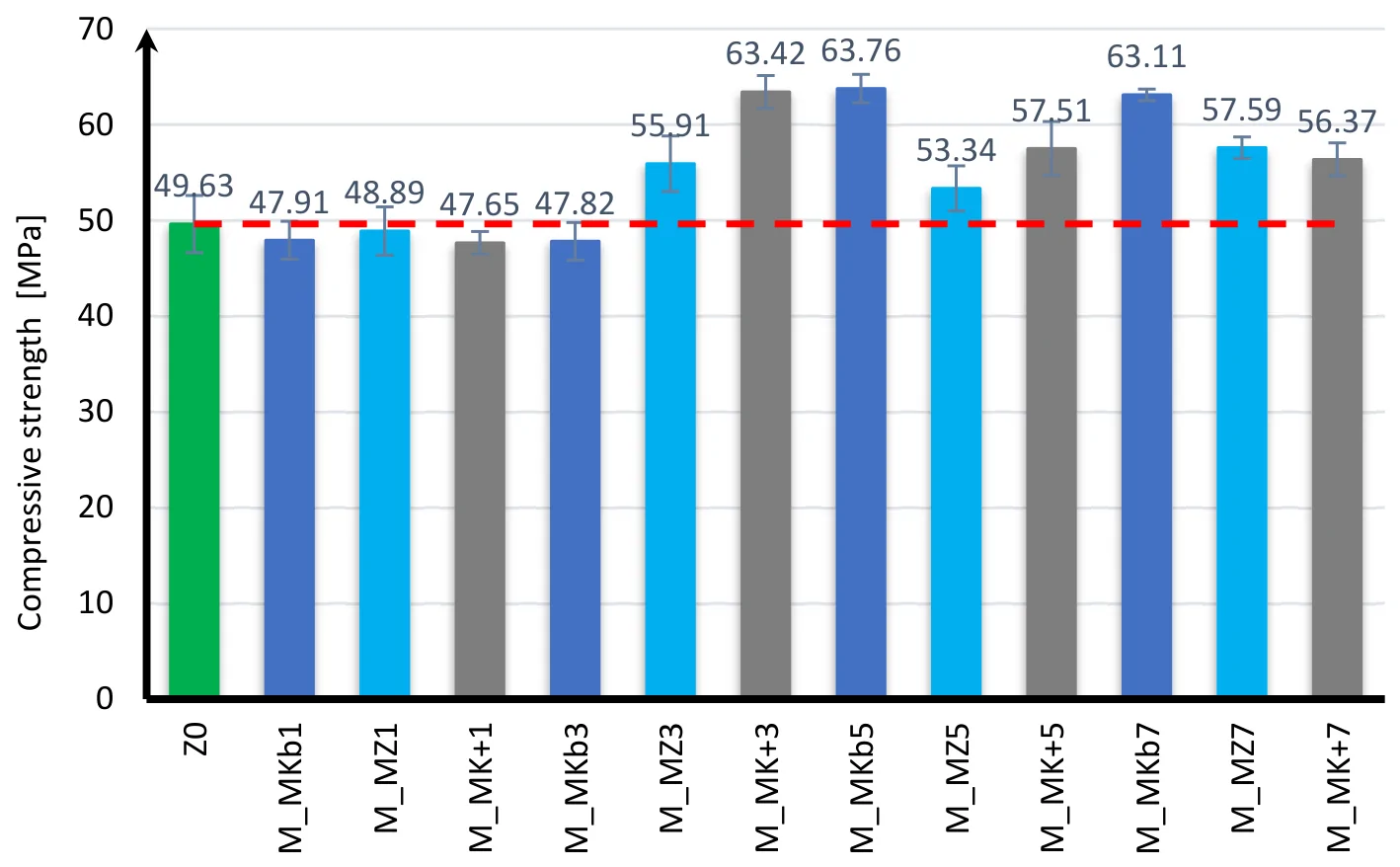
Compressive strength after 56 days of conditioning for samples with the addition of microsilica in dry form.

**Figure 22:**
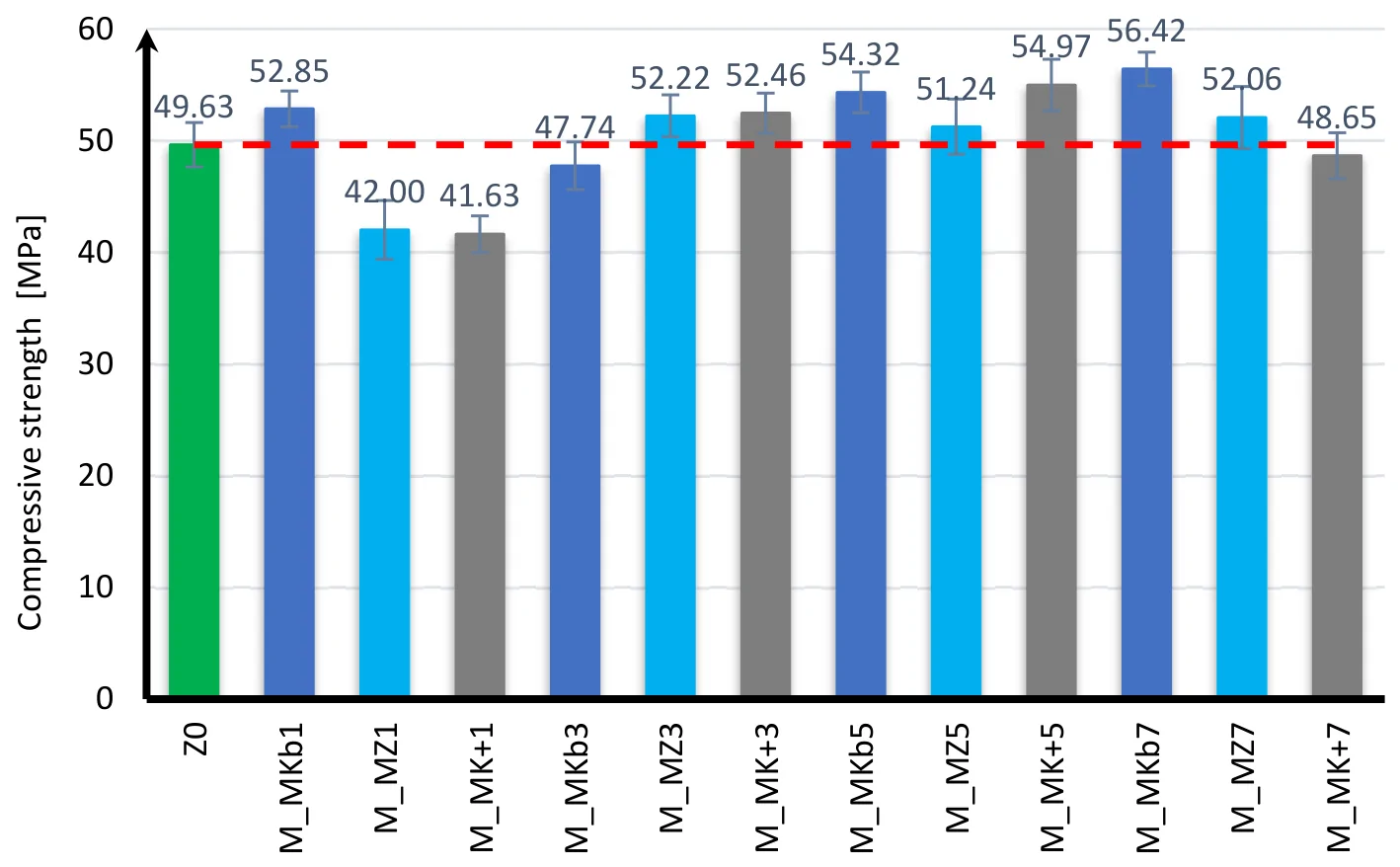
Compressive strength after 56 days of conditioning for samples with the addition of microsilica in the form of a suspension.

The compressive strength of the mortar was tested in the same manner as for the bending strength test after 7, 28, and 56 days of sample conditioning. The compressive strength test results after 7 days of conditioning are presented in Figure 17 and Figure 18, those after 28 days of conditioning are presented in Figure 19 and Figure 20, and those after 56 days of conditioning are presented in Figure 21 and Figure 22. The figures show the mean compressive strength results obtained from measurements of 18 specimens per series, along with the standard deviation.

When analyzing the compressive strength results of the mortar samples after 7 days of conditioning, it can be concluded that, unlike bending strength, adding microsilica in suspension had a positive effect on the strength results, while adding dry microsilica had a negative effect on the strength results after 7 days of conditioning. With dry microsilica, most samples achieved similar results, ranging from approximately 32 MPa to 35 MPa. The highest strength was achieved by samples with a 1% addition of white microsilica, whose compressive strength was 9% lower than those of the base samples. The lowest strength was achieved by samples with 3% compacted microsilica. Their strength was as much as 30% lower than the strength of samples without additives. The remaining samples had strengths ranging from 17% to 25% lower than those of the base samples. In the case of the addition of microsilica in the form of a suspension, six batches were characterized by strengths 10% to 15% lower than those of the base samples, five batches achieved results similar to the samples without additives, and only one batch, with a 1% addition of white microsilica, achieved compressive strength 4% higher than those of the base samples. Based on the presented strength results, it is not possible to clearly determine the dependence of the compressive strength after 7 days of conditioning on the content of individual microsilicas.

An analysis of the compressive strength results after 28 days of curing reveals a greater strength gain in samples containing dry microsilica compared to those containing microsilica in the form of an aqueous suspension. In the case of dry microsilica, the highest strength was achieved by samples with 1% white microsilica, which was 4% higher than the strength of the samples without additives. Samples with 3% Mikrosill+ microsilica and 5% white microsilica achieved compressive strength equivalent to the samples without additives. Samples with 5% compacted and Mikrosill+ microsilica and those with 7% concentrated white and Mikrosill+ microsilica showed results similar to the baseline samples. Their strength was 4% to 8% lower than the strength of the samples without additives. The lowest strength was observed in samples with 1% compacted and Mikrosill+ microsilica and those with 3% white and compacted microsilica. Their strength was 10% to 17% lower than the strength of the base samples. When microsilica was added as an aqueous suspension, its effect on the compressive strength results after 28 days was similar to that after 7 days of conditioning. Only samples with 5% white and Mikrosill+ microsilica achieved compressive strengths that were 9% and 10% higher, respectively, than the base samples. Samples with 7% white microsilica have a strength 5% higher than those of the base samples, while samples with 1% white microsilica, 3% Mikrosill+ microsilica, 5% compacted microsilica, and 7% compacted microsilica have results comparable to those without additives. Samples with 1% compacted and Mikrosill+ microsilica and those with 3% white microsilica have the lowest strength. Their strengths are 14% to 17% lower than the strength of the samples without additives.

In the case of compressive strength results after 56 days of conditioning, most samples with both dry and aqueous microsilica additions had a positive effect on strength. When analyzing the results for dry microsilica additions, it can be concluded that samples with 1% white, compacted, and Mikrosill+ microsilica and those with 3% white microsilica achieved compressive strength results slightly lower than the samples without additions. Their strengths are 1% to 4% lower than those of the base samples. The remaining samples exhibit strengths clearly higher than those of the base samples. Among them, the highest strengths are achieved by samples with 3% Mikrosill+ microsilica and 5% and 7% white microsilica. Their strengths are 28% higher than those of the base samples. The remaining samples exhibited strengths higher than those of the base samples, ranging from 7% for a 5% compacted microsilica content to 16% for a 7% compacted microsilica content. When microsilica was added as an aqueous suspension, the lowest strengths were found in samples with 1% compacted and Mikrosill+ microsilica. Their strengths were 15% and 16% lower than the base samples, respectively. The highest strengths were found in samples with 5% white and Mikrosill+ microsilica and 7% white microsilica. Their compressive strengths were 9% to 14% higher than those of the samples without additives. The remaining samples achieved compressive strengths similar to those of the samples without additives, with the samples with a 3% white microsilica content and a 7% Mikrosill+ microsilica content having strengths approximately 3% lower than those of the base samples.

**Figure 23:**
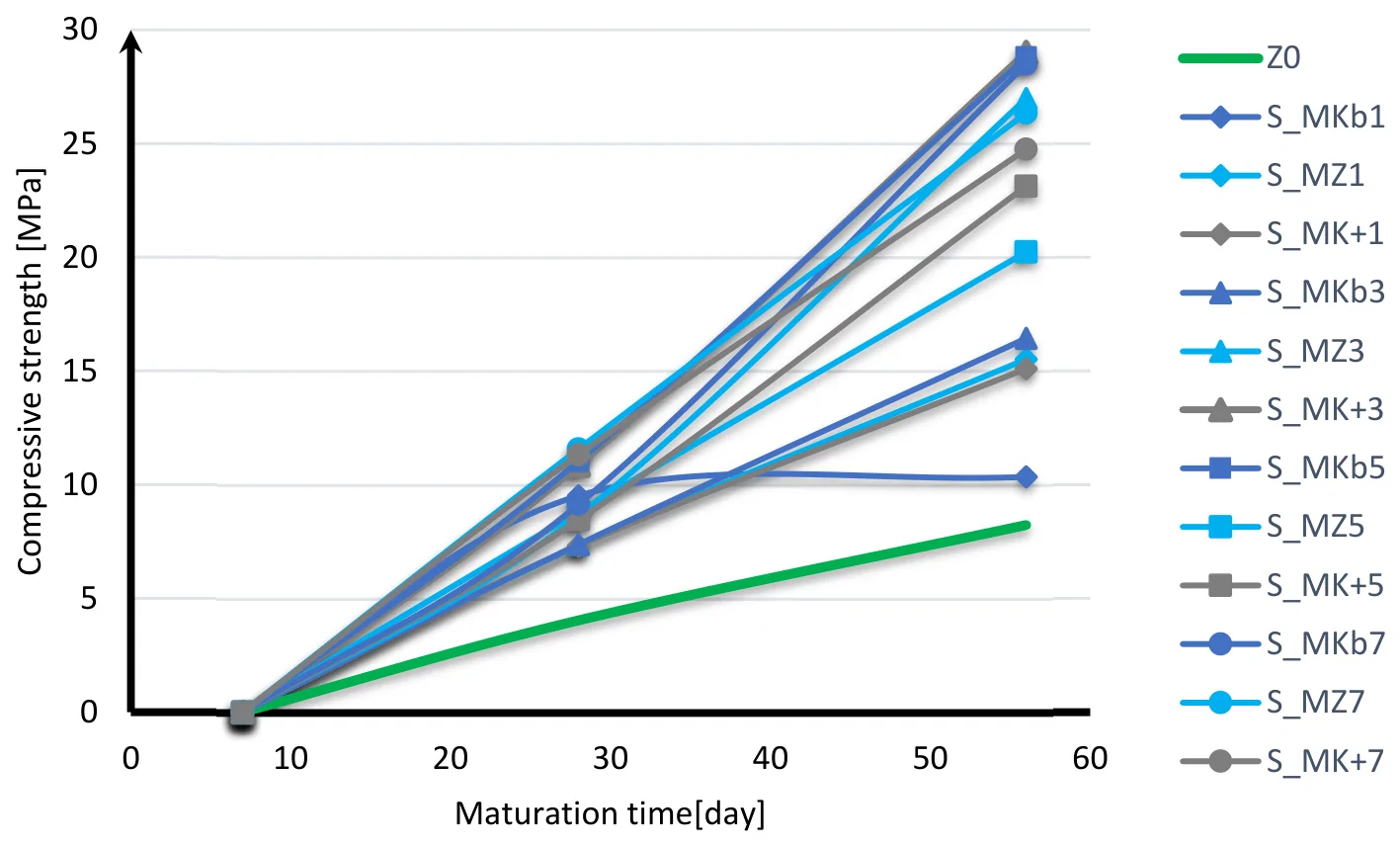
The increase in compressive strength over time for samples with the addition of dry microsilicas.

**Figure 24:**
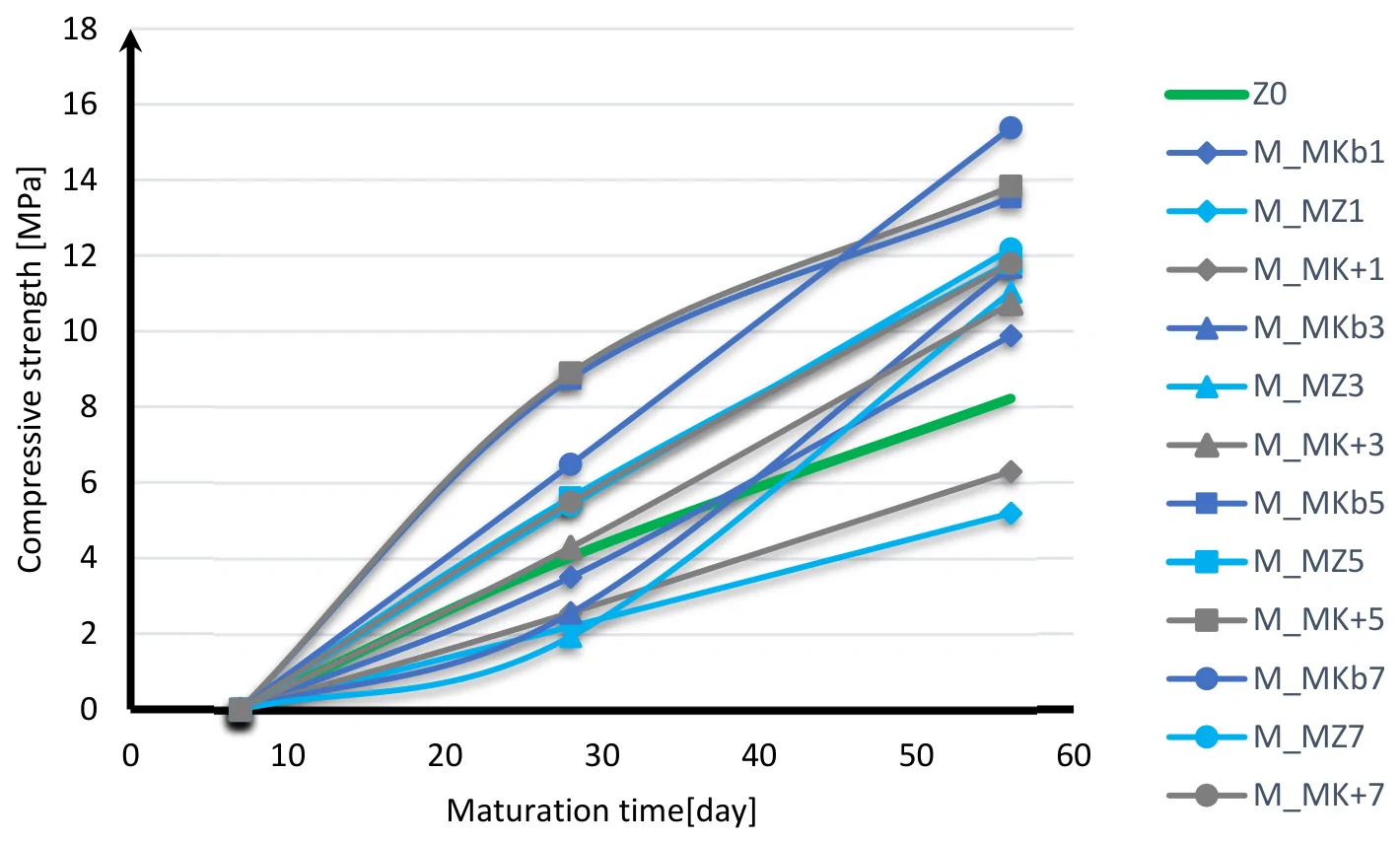
The increase in compressive strength over time for samples with the addition of microsilica in the form of a suspension.

When analyzing the graphs showing increases in compressive strength for microsilica added in dry form, shown in Figure 23, and for microsilica added as an aqueous suspension, shown in Figure 24, it can be concluded that in most cases, the addition of microsilica has a beneficial effect, causing a faster increase in strength over time compared to samples without microsilica. For microsilica added in dry form, all samples are characterized by faster early strength increase between 7 and 28 days and late strength increase between 28 and 56 days compared to the baseline samples. It can also be observed that all samples, except for the sample with 1% addition of white microsilica, are characterized by a linear strength increase over time. The greatest increase in strength between days 7 and 28 of conditioning was observed for 3% Mikrosill+ microsilica, 5% white microsilica, and 7% compacted and Mikrosill+ microsilica. The greatest increase in strength between days 28 and 56 of conditioning was observed for samples with 3% compacted and Mikrosill+ microsilica, 5% white microsilica, and 7% white and compacted microsilica. When microsilica was added as an aqueous suspension, not all samples showed a greater increase in strength compared to the base samples. When analyzing the increase in strength between days 7 and 28 of conditioning, it should be noted that samples with 3% Mikrosill+ microsilica and all samples with 5% and 7% microsilica addition showed a greater increase in strength than the base samples. The greatest increase among them was observed in samples with a 5% content of white microsilica and Mikrosill+, which was twice as high as in the base samples. The remaining samples showed a smaller increase in strength compared to the base samples. When analyzing the strength increase between days 28 and 56 of conditioning, it can be concluded that all samples, except those with a 1% content of compacted and Mikrosill+ microsilica, showed a greater increase in strength than the samples without additives. The greatest increase in strength was observed in samples with a 5% content of white and Mikrosill+ microsilica and in those with a 7% content of white microsilica. Comparing the results of the strength increase between microsilicas added in dry form and in the form of an aqueous suspension, it can be stated that dry microsilicas are characterized by a 1.5 to 2 times greater increase in strength both between the 7th and 28th days of conditioning and between the 28th and 56th days of conditioning than the samples with the addition of microsilicas in the form of an aqueous suspension.

## 4. Discussion

The results obtained in this article show that the effect of microsilica dosing method on the properties of standard mortar depended on the type of microsilica, its content, conditioning time, and the analyzed property. No clear advantage was observed between dry dosing or dosing as an aqueous suspension in all tested mixtures. This means that the method of introducing microsilica into the standard mortar should not be considered independently of physical properties.

While the obtained consistency results show that individual microsilicas and their content have a direct impact on consistency, it is impossible to clearly determine the advantage of one dosing method over the other. The results showed similar consistency values between the dosing methods for half of the samples, while for the other half, differences of up to 50% were observed. However, no correlation was observed between the content of individual microsilicas and the discrepancies in the obtained results.

Bending strength results showed a clear advantage for samples with dry-dosed microsilica after 7 and 28 days of curing. After 7 days, all samples with microsilica dosed as a slurry achieved values lower than the base sample, while many samples with the dry additive achieved values higher than the base sample. However, this relationship was not unequivocally maintained after 56 days. Of particular interest is the fact that slurry dosing, which, from a technological perspective, can promote the initial dispersion of particles in water, did not automatically lead to an increase in bending strength. After 56 days, the effect of dosing method was less clear. For some mixtures, the differences between the methods were small, below 10%, while in two cases, i.e., for 3% compacted microsilica and 7% compacted microsilica, a difference of 19% was obtained. At the same time, most samples had lower strength than those of the reference samples. In this case, it is also impossible to clearly determine which method of dosing microsilica was more advantageous, while for both methods, samples with 5% white microsilica showed the highest strength. However, it can be observed that in these samples, dry dosing was characterized by a greater increase in early strength compared to the wet dosing of microsilica. Based on the bending strength results, it is impossible to clearly determine the beneficial effect of microsilica or its dosing method. However, it should be noted that microsilica added in the form of a suspension was characterized by a more stable increase in strength over time compared to samples with dry microsilica. Without conducting more extensive research analyzing the maturation process of individual composites, the cause of this phenomenon cannot be clearly determined.

In the case of compressive strength, a different relationship was observed than that observed for bending strength. After 7 days, the microsilica dosage in the form of an aqueous suspension was more favorable in most of the tested variants. Differences compared to the corresponding samples with a dry additive ranged from approximately 10% to 42%. The largest differences were observed for compacted microsilica, particularly at 3% and 7%. After 28 days, the differences between the dosage methods were smaller. Only in the case of the 5% content of Mikrosill+ microsilica were more favorable results for the suspension obtained, for which the difference exceeded 10%, while for the other values, they were similar. This indicates a gradual decrease in the influence of the initial microsilica preparation method as the curing process progressed. A similar trend was observed after 56 days of conditioning. In this case, samples with a minimum 3% microsilica content exhibited higher strengths. The effect of dosing method was observed for samples with a 3% Mikrosill+ microsilica content and 5% and 7% white microsilica contents. When analyzing all samples, it can be concluded that the samples with dry microsilica dosing performed slightly better overall than the baseline samples. Furthermore, the use of dry microsilica guarantees a certain increase in strength over time, which is greater compared to the reference samples. However, the use of microsilica in suspension resulted in an uneven increase in strength over time. This means that in the tested system, a universal relationship cannot be assumed according to which the use of microsilica in aqueous suspension leads to greater strength. Rather, the results indicate a relationship between the dosing method, the type of microsilica, and the mortar’s curing age. This is consistent with the authors’ results presented in [7,32], where they analyzed the effect of compacted microsilica and Mikrosill on the properties of a standard mortar made from CEM I 42.5R cement and quartz sand. The authors demonstrated that the use of microsilica can lead to increased compressive strength, with the effect depending on the type and content of the additive. When 10% of the cement was replaced with microsilica, the increase in compressive strength exceeded 40% compared to the reference mortar.

Similar conclusions were reached by the authors of [30] who also did not demonstrate a universal advantage of the microsilica dosing method, which is consistent with the observation that the suspension dosing method does not guarantee the achievement of better mechanical properties.

The tested materials exhibited distinctly different behavior upon contact with water. Compacted microsilica exhibited a relatively stable granulometric distribution, independent of water conditioning time. The particle size distribution of Mikrosill+ microsilica was influenced by the duration of water conditioning, with the disintegration of agglomerates observed as the time increased. White microsilica, on the other hand, exhibited a particularly strong tendency to form particle clusters, especially after short-term water conditioning, and then disintegrate after 210 hours of water conditioning. At the same time, SEM images indicated the presence of much finer primary particles. The discrepancy between these results can be interpreted as the presence of secondary agglomerates, which are detected by granulometric analysis, while SEM allows for the observation of their components. This is consistent with the results of the authors of [30], who analyzed the dispersion of raw, moderately compacted, and highly compacted microsilica in water and cement slurry filtrate. The authors demonstrated clear differences in the degree of agglomeration between the tested materials. D50 for RSF, MDSF, and HDSF was approximately 10.9, 15.0, and 112.2 µm, respectively, indicating a very strong effect of the degree of densification on the state of agglomeration. It is also important to note that contact with water did not lead to the same behavior in all materials. The obtained results do not confirm the universal mechanism according to which contact of microsilica with water always leads to its deagglomeration. Depending on the type of material, water may reduce agglomeration, temporarily increase it, or create a system with a more complex structure. Therefore, the suspension dosing method should not be automatically associated with better dispersion. The differences between MKb, MZ, and MK+ are particularly important in the context of the obtained mechanical results. The authors of [22] compared raw and compacted microsilica in pastes, mortars, and concretes. The authors demonstrated that raw microsilica was characterized by better dispersion in the paste than compacted microsilica, while these differences were smaller in the case of concrete. At the same time, both microsilica varieties led to increased strength, and the effect of the additive depended on the type of system tested.

Based on the conducted research, it can be concluded that the effectiveness of microsilica in mortar depends on the simultaneous influence of several factors: chemical composition, grain size characteristics, specific surface area, tendency to form agglomerates, dosing method, additive content, and curing time. The results do not support the hypothesis of a universal advantage of the suspension dosing method. The effect of this method depended on the type of microsilica and its mechanical properties. Slurry dosing was more beneficial in many cases for early compressive strength, while dry dosing was more often beneficial for bending strength at 7 and 28 days. After 56 days, the advantage of dry dosing was particularly evident in the case of compressive strength. Granulometric results also indicate that contact with water did not lead to a uniform change in the agglomeration state of all the materials analyzed. Therefore, the dosing method should be considered not as an independent technological factor, but as an element affecting a specific material with specific physical and chemical properties.

## 5. Conclusions

1. The use of microsilica affected the consistency of the standard mortar, with the direction and magnitude of changes depending on the type of microsilica and its content. The effect of the dosing method alone was relatively small for most mixtures, although more pronounced differences were observed for selected low MKb and MZ contents.
2. In the case of bending strength, better results were generally obtained after 7 and 28 days for dry-dosed microsilicas. After 56 days, the effect of dosing method was less clear and depended on the type of microsilica and its content.
3. Compressive strength varied depending on the dosing method. After 7 days, in most cases, better results were obtained for microsilica added as an aqueous suspension. After 56 days, in most variants, higher strength was achieved for samples with microsilica dosed dry.
4. The highest compressive strength values after 56 days were obtained for selected mixtures with dry-dosed microsilica, in particular for S_MKb5, S_MKb7 and S_MK+3. These values were approximately 63.76, 63.11 and 63.42 MPa, respectively, compared to 49.63 MPa for the base sample, Z0.
5. The type of microsilica influenced the obtained results. MKb, MZ, and MK+ differed in chemical composition, specific surface area, and agglomerate formation, and these differences were reflected in the mortars’ response to the dosing method.
6. Granulometric analysis revealed different behavior of the tested microsilicas during contact with water. MKb showed a strong tendency to form secondary agglomerates, MK+ was characterized by dynamic changes in the share of larger agglomerates, while MZ showed a relatively stable granulometric distribution.
7. No universal advantage was found between dry and slurry dosing of microsilica. The effect of the dosing method depended on the type of material, its content, age of maturation, and the type of mechanical property being tested.
8. The results indicate that the microsilica dosing method should not be directly associated with a specific degree of deagglomeration. Contact with water caused different changes in particle size distribution depending on the type of microsilica. Further microstructural and hydration studies are required to clearly explain the mechanism.

## Figures and Tables

**Table 1:** Properties of the CEM I 42.5 R cement used in the study.

Property	Unit	Value
Specific surface area	cm^2^/g	4124
Start of setting	Min	184
End of setting	Min	242
Volume change	Mm	1.0
Compressive strength		
After 2 days	MPa	30.1
After 28 days	MPa	60.2
SO_3_ content	%	2.95
Cl content	%	0.089
Insoluble residue	%	0.57
Loss on ignition	%	3.33

**Table 2:** Chemical composition of the additives used.

Component	Unit	MKb	MZ	MK+
SiO_2_	%	>94.0	>80.0	>85.0
CaO	%	<1.0	<3.5	<1.0
ZrO_2_	%	<4.0		
Fe_2_O_3_	%	<1.0		
SO_3_	%		<4.0	<2.0
Na_2_O	%		<8.0	<0.5
Al_2_O_3_	%	<1.0		
Cl^−^	%	<1.8	<1.8	<0.3

**Table 3:** Grain size parameters of the microsilicas used.

Property	Unit	MKb	MZ	MK+	CEM I
SS Dry	cm^2^/g	36,540	75,224	84,843	4124
SS Water 210h	cm^2^/g	46,250	93,050	98,677	-
D90 Dry	μm	1015.24	42.84	34.69	56.87
D90 Water 210h	μm	1006.72	40.79	15.38	-
D50 Dry	μm	718.35	21.40	12.36	16.33
D50 Water 210h	μm	711.31	19.21	0.29	-

**Table 4:** Composition of mixtures.

Scheme 0	Type of Supplement	Sand Content [g]	Cement Content [g]	Contents of the Supplement [g]	Water Content [g]
Z0	-	1350	450	-	225
S_MKb1	Dry white microsilica	1350	445.5	4.5	225
S_MKb3		1350	436.5	13.5	225
S_MKb5		1350	427.5	22.5	225
S_MKb7		1350	418.5	31.5	225
S_MZ1	Dry compacted microsilica	1350	445.5	4.5	225
S_MZ3		1350	436.5	13.5	225
S_MZ5		1350	427.5	22.5	225
S_MZ7		1350	418.5	31.5	225
S_MK+1	Dry Mikrosill+ microsilica	1350	445.5	4.5	225
S_MK+3		1350	436.5	13.5	225
S_MK+5		1350	427.5	22.5	225
S_MK+7		1350	418.5	31.5	225
M_MKb1	Wet white microsilica	1350	445.5	4.5	225
M_MKb3		1350	436.5	13.5	225
M_MKb5		1350	427.5	22.5	225
M_MKb7		1350	418.5	31.5	225
M_MZ1	Wet compacted microsilica	1350	445.5	4.5	225
M_MZ3		1350	436.5	13.5	225
M_MZ5		1350	427.5	22.5	225
M_MZ7		1350	418.5	31.5	225
M_MK+1	Wet Mikrosill+ microsilica	1350	445.5	4.5	225
M_MK+3		1350	436.5	13.5	225
M_MK+5		1350	427.5	22.5	225
M_MK+7		1350	418.5	31.5	225

## Data Availability

The original contributions presented in this study are included in the article. Further inquiries can be directed to the corresponding author.
